# Understanding building damage through the lens of the Swiss post-seismic reconnaissance mission of 2023 Al Haouz, Morocco, earthquake

**DOI:** 10.1038/s41598-025-00659-2

**Published:** 2025-05-13

**Authors:** Afifa Imtiaz, Savvas Saloustros, Meriton Beqiraj, Gustavo Cortés, Mylène Devaux, Eric Lattion, Yuhan Zhu, Hamza Sehaqui

**Affiliations:** 1 Swiss Seismological Service (SED), ETH Zurich, Sonneggstrasse 5, NO H55, CH-8092 Zurich, Switzerland; 2 Earthquake Engineering and Structural Dynamics laboratory (EESD), School of Architecture, Civil and Environmental Engineering (ENAC), École Polytechnique Fédérale de Lausanne (EPFL), Lausanne, Switzerland; 3 Résonance Ingénieurs-Conseils SA, Carouge, Switzerland; 4 Exigo Expertises SA, Ecublens, Switzerland; 5 Institute of Construction and Environmental Technologies (iTEC), Haute Ecole d’Ingénierie et d’Architecture de Fribourg (HEIA), University of Applied Sciences and Art of Western Switzerland (HES-SO), Delémont, Switzerland; 6 Lattion Bruchez Ingénieurs SA, Collombey-Muraz, Switzerland; 7 Editech SA, Ayent, Switzerland

**Keywords:** Earthquake reconnaissance, Earthquake damage, Slowly deforming mountain belts, Morocco Al Haouz earthquake, Reinforced concrete structures, Masonry structures., Natural hazards, Civil engineering, Seismology

## Abstract

A team of scientists and engineers from Swiss institutions participated in a post-seismic reconnaissance mission in Morocco following the magnitude 6.8 earthquake of September 8, 2023. We visited different heavily affected towns and villages, located from 10 to 70 km epicentral distance, in the High Atlas Mountain and Marrakech. In this work, we report our observations from inspecting building damage in affected areas. We discuss the prevalent building typologies observed, their construction mechanisms, and the resulting structural and non-structural damage patterns. We also examine the potential site-related effects based on a literature review of the seismological and geological settings of the area. The earthquake’s severe impact was due to its shallow depth and underlying geological complexities, including active faulting and diverse rock formations. The lack of earthquake-resistant construction practices significantly exacerbated the damage. Modern structures in Marrakech were largely unaffected while ancient ones in the Medina of Marrakech suffered partial damage. In rural High Atlas areas, buildings exhibited significant damage due to lack of seismic design as well as poor-quality materials. Our observations prompt us to believe that conducting site-specific hazard studies along with implementing earthquake-proofing measures involving local communities can foster resilience to future seismic events in this area.

## Introduction

Slowly deforming mountain belts can experience damaging earthquakes, though these events are less frequent and intense than those at active plate boundaries. The rarity of these events often results in low hazard awareness among communities, leading to insufficient preparation and a lack of earthquake-resistant construction practices. Observational studies in areas affected by these events provide valuable insights into the factors contributing to high damage and fatalities, particularly in remote mountainous regions.

**Fig. 1:**
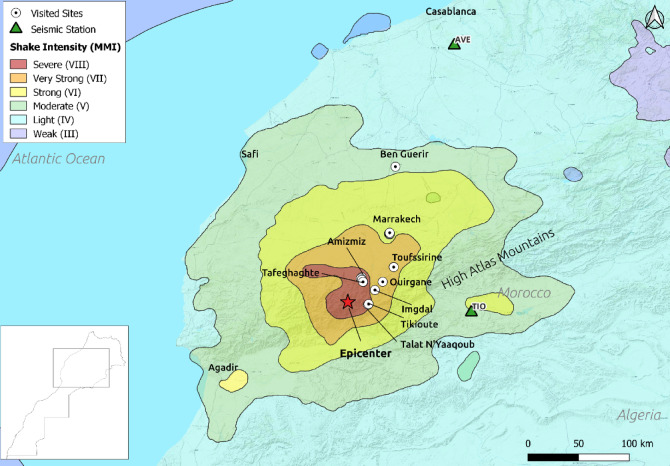
Map of ground shaking intensity in terms of MMI (Modified Mercalli Intensity) of the 2023 Al Haouz earthquake (reported by the USGS) showing sites visited by the SGEB team. The contours of macroseismic intensity have been plotted from the JSON file of ‘Intensity Contours’ provided by the USGS^2^. Produced by QGIS (version Desktop 3.34.15); https://qgis.org/.

On September 8, 2023, at 11 p.m. local time (UTC 22:11), a moment magnitude (Mw) 6.8 earthquake struck Morocco. According to the United States Geological Survey (USGS) ^23^, it occurred at a shallow depth of 19 km in the Al Haouz province, about 500 km from the North African and Eurasian plate boundary, an area of low seismic activity. A magnitude (M) 4.9 aftershock followed 20 min later, worsening the damage. The Interior Ministry of Morocco reported over 2,900 fatalities and 5,600 injuries, with significant building damage and losses in mountainous villages near the epicenter. Figure 1 shows the ground-shaking footprint based on the USGS observations from seismic stations and damage reports (1).

Understanding the seismic perils of an event like the Al Haouz, Morocco, earthquake is of great interest to the earthquake engineering community of Switzerland. The country experiences low to moderate seismic activity, with larger but infrequent events occurring within an intra-plate setting. The strongest documented earthquake in Switzerland’s history is the Mw 6.6 event of 1356. The event, centered near Basel, caused widespread damage to buildings in northwestern Switzerland and was felt across much of Europe. To reduce the potential impact of a repeat of such an event, the federal authorities in Switzerland have been implementing an earthquake risk mitigation program over the last two decades. In this light, the Swiss Society for Earthquake Engineering and Structural Dynamics (SGEB) sent a team to conduct a post-seismic reconnaissance mission in Morocco. The current study aims to summarize observations from this mission.

The mission took place between November 23 and 27, 2023. The team, consisting of scientists and engineers from different Swiss institutes, visited different heavily affected towns and villages (Fig. 1), located between 10 and 70 km epicentral distance (R), namely Talat N’Yaaqoub (*R* = 11 km), Tikioute (*R* = 11 km), Imgdal (*R* = 15 km), Tafeghaghte (*R* = 15 km), Amizmiz (*R* = 18 km), Baraka (*R* = 25 km), Toufssirine (*R* = 42 km), and Marrakech (*R* = 70 km).

Details of the mission are provided in the reconnaissance report^3^. In this work, we focus on our main observations on the potential of site-related effects, prevalent building typologies observed, their construction mechanism, and consequent structural and non-structural damage patterns observed. We hope that the valuable insights we have gained through our mission will not only benefit Switzerland but also inform the global scientific community about the seismic risks in slowly deforming mountain belts.

## Seismological aspects

### Seismicity and seismotectonics of Morocco

Morocco, located at the convergence of Africa and Europe, features a complex tectonic framework shaped by interactions between major tectonic plates. Cheloni et al.^4^ describe three primary structural domains: the Rif, the Atlas Mountains, and the Anti-Atlas. The northward convergence of the African (Nubian) plate with the Eurasian plate at 4–10 mm per year has formed the Rif mountain range and the Strait of Gibraltar, making northern Morocco prone to significant seismic activity. The Atlas Mountains in the south were initially formed from the breakup of Pangea in the early Jurassic period and were uplifted during the Cenozoic period due to ongoing collisions of the North African and Eurasian plates. This collision causes active deformation in the High Atlas, with GPS data showing a shortening rate of about 1 mm/yr^5^. This results in faults with reverse and strike-slip motion, consistent with the focal mechanism of the Al Haouz event. However, the slower deformation rate means stress accumulation requires longer periods compared to the north to generate similar magnitude earthquakes.

Historical records show Morocco has experienced significant earthquakes, such as those in Fez (1624) and Agadir (1731). South of the Mediterranean, seismic activity is less frequent and of smaller magnitude. However, significant events like the 1960 Agadir earthquake (Mw 5.8) have occurred, causing widespread destruction. The region’s seismicity is driven by a complex network of active faults with strike-slip or reverse mechanisms and potential lithosphere thinning combined with mantle uplift^5^, highlighting the ongoing risk of large magnitude earthquakes.

### Observed ground motion

The USGS reported ground shaking intensities V to VI on the MMI scale, indicating moderate to strong tremors and slight damage, within a radius of approximately 150 km from the epicenter of the Al Haouz event. Closer to the epicenter, intensity VIII was observed, indicating severe shaking with heavy damage. In Marrakech, intensities reaching up to VI were observed, the level at which structural damage starts to appear in non-engineered structures. The North African hazard map provided by Poggi et al.^6^ estimates a peak ground acceleration (PGA) of 0.13–0.2 g in Marrakech for the 475-year return period. The PGA footprint of USGS shows a ground motion level between 0.1 and 0.2 g, reaching up to 0.5 g at some observation points in the city. So, the observed ground motion seems to have exceeded the 475-year hazard level.

**Fig. 2:**
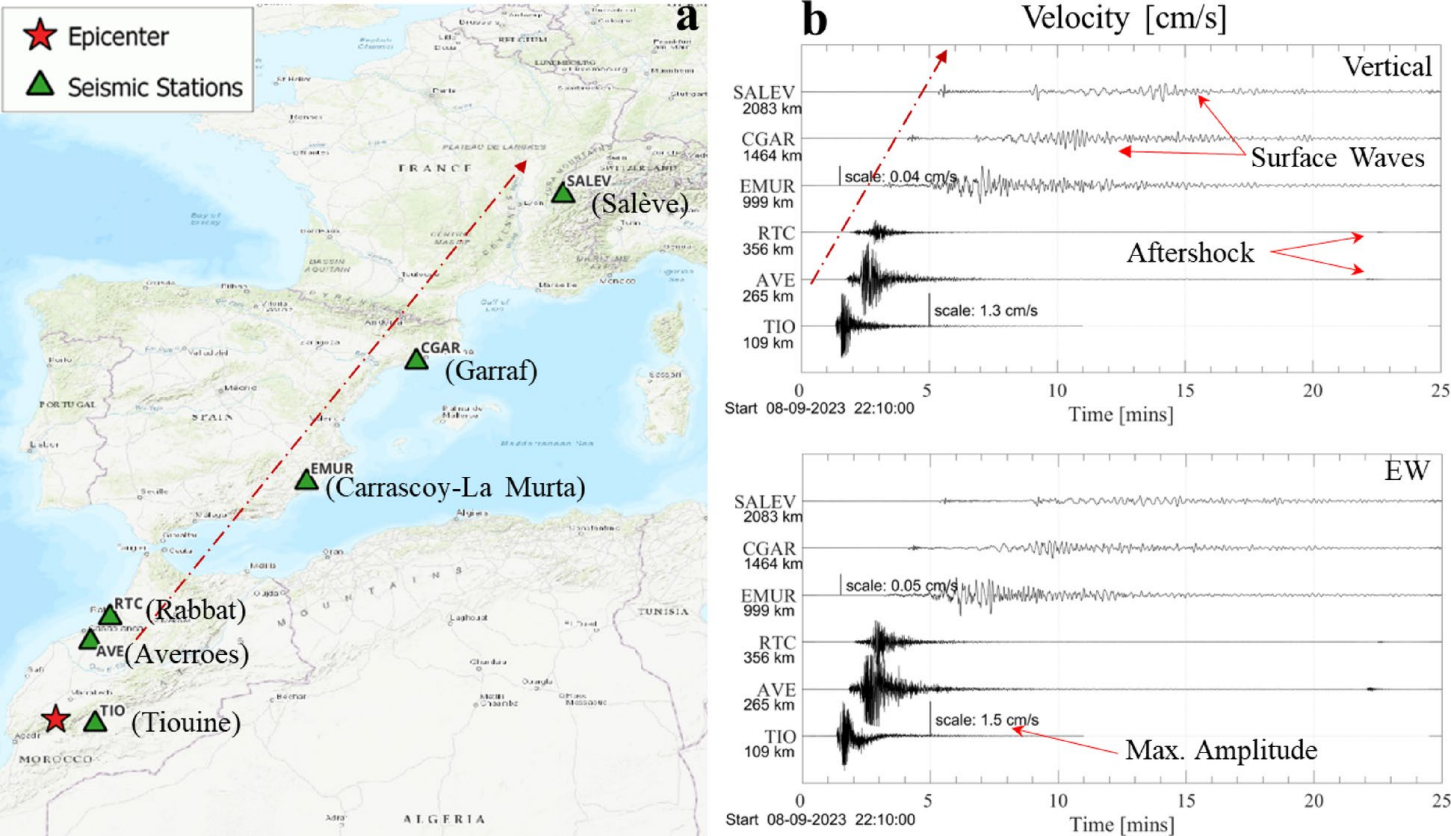
(**a**) Seismic stations with publicly available waveforms from 2023 Al Haouz event. Produced by QGIS (version Desktop 3.34.15); https://qgis.org/. (**b**) Velocigrams (cm/s) from vertical and EW components. Recordings from stations in and outside Morocco are plotted on different amplitude scales for better visibility. Produced by MATLAB (version R2024b); https://ch.mathworks.com/products/matlab.html/.

Figure 2 (a) shows the locations of several stations (see Table 1) with publicly available data from European networks. Seismograms of one horizontal (east-west, EW) and the vertical components are displayed in Fig. 2 (b). The nearest station Tiouine (TIO), situated approximately 100 km away from the epicenter, experiencing a moderate ground shaking (see Fig. 1), recorded a peak ground velocity (PGV) of 1.5 cm/s.

**Table 1:** List of seismic stations with available data.

Network code	Station code	Station description	Epicentral distance (km)	Reference
WM	TIO	ROA/UCM/GEOFON Station Tiouine, Morocco	109	WMSN (2023)^7^
WM	AVE	ROA/UCM Station Averroes, Morocco	265	WMSN (2023)^7^
MN	RTC	Rabat, Morocco	356	MVBSN (2023)^8^
ES	EMUR	La Murta (Murcia) IGN, Spain	999	SDSN (2023)^9^
CA	CGAR	Garraf, Spain	1464	CSN (2023)^10^
CH	SALEV	Salève, Haute-Savoie, France	2083	SSN (2023)^11^

**Fig. 3:**
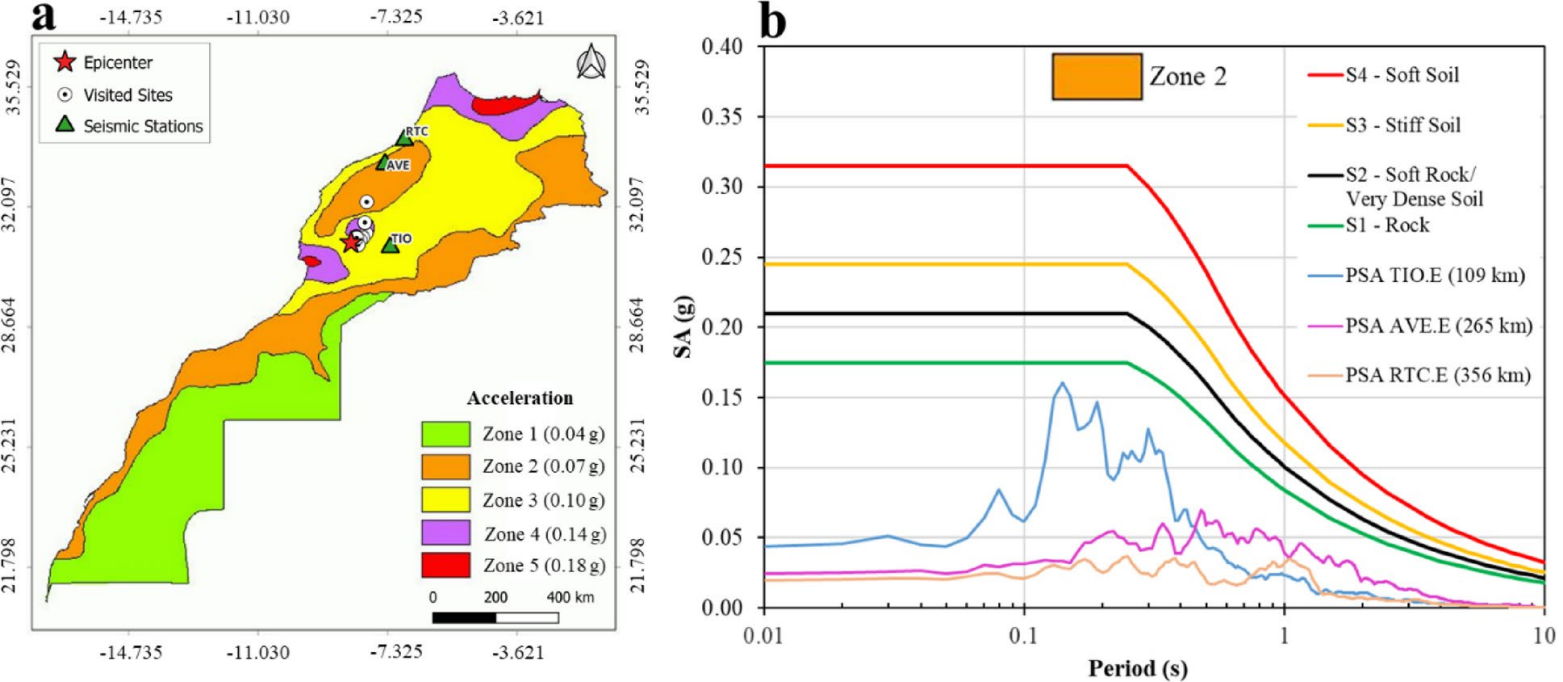
(**a**) National seismic standard of Morocco (RPS2000, 2011)^12^ with acceleration on rock for probabilities of 10% in 50 years. Produced by QGIS (version Desktop 3.34.15); https://qgis.org/. (**b**) Comparison between the normalized response spectra of the RPS Zone 2, after applying the amplification factors, and PSA spectra estimated from EW component of seismic recordings of the stations located in Morocco. Produced by MATLAB (version R2024b); https://ch.mathworks.com/products/matlab.html/.

Since 2004, Morocco has enforced a national seismic standard RPS2000, revised in 2011^12^. It shows acceleration coefficients with a 10% probability of exceedance over 50 years (a return period of 475 years), based on PGA values for the Design Basis Earthquake (DBE) hazard level (Fig. 3a). The sites visited by the SGEB team fall within low to moderately seismic zones 2 and 4, with DBE PGA values of 0.07 g to 0.14 g. We compare the pseudo-spectral acceleration (PSA) at 5% damping derived from three seismic stations in Morocco (TIO, AVE, RTC in Table 1) with the RPS spectra for various soil types in Zone 2, after considering the site amplification factors prescribed by the code (Fig. 3b).

Notably, Tiouine (TIO) station displays a spectral acceleration peak of 0.16 g at a period of 0.14 s, even though it is located far away (109 km) from the epicenter. The locations visited by the SGEB team lie within the 0.2 to 0.5 g PGA levels (reported by USGS, 2023). In the near-fault areas, this level of seismic ground motions contains large velocity pulses that can increase the displacement demand along the full height of the structures^13^.

### Signs of potential site effects

The event caused significant structural damage within a 60 km radius from the epicenter, especially affecting smaller communities in the Atlas Mountains’ foothills. Several factors including the earthquake’s magnitude and shallow depth, the complex network of faults (see Cheloni et al.^4^, subsequent aftershocks (see Bao et al.^14^, and aging buildings lacking adherence to seismic design contributed to the damage. The areas we explored predominantly lie in the western High Atlas outcrops and the Haouz basin. The High Atlas’s complex geological structure, with folded and faulted rock formations, affects ground motion and seismic wave transmission, potentially causing localized amplification. Rugged topography, characterized by steep slopes and deep valleys, can focus and amplify seismic energy. Sedimentary basins with varying seismic velocities compared to solid bedrock can trap and amplify seismic energy, leading to stronger ground shaking. Additionally, resonance effects, where the natural frequency of the subsurface layers matches the seismic waves’ frequency, can increase the amplitude and duration of ground motion, causing severe damage when these frequencies align with those of buildings.

**Fig. 4:**
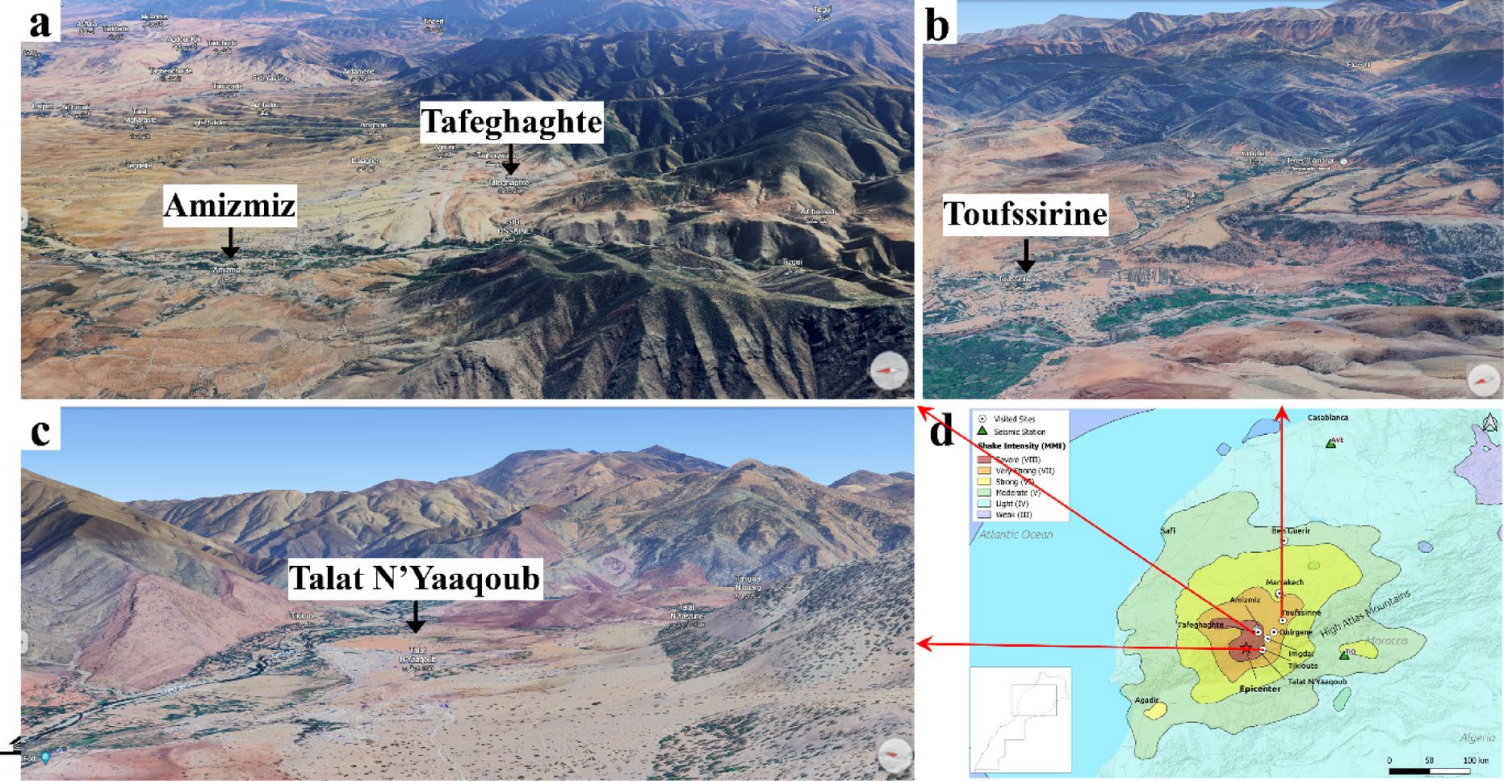
(**a**–**c**) Topographic images of some of the visited sites (GE3D^15–17^. Produced by Google Earth (version 10.49.0.0 online); https://earth.google.com/web/. (**d**) Locations of sites on the shake-map from Fig. 1.

**Fig. 5:**
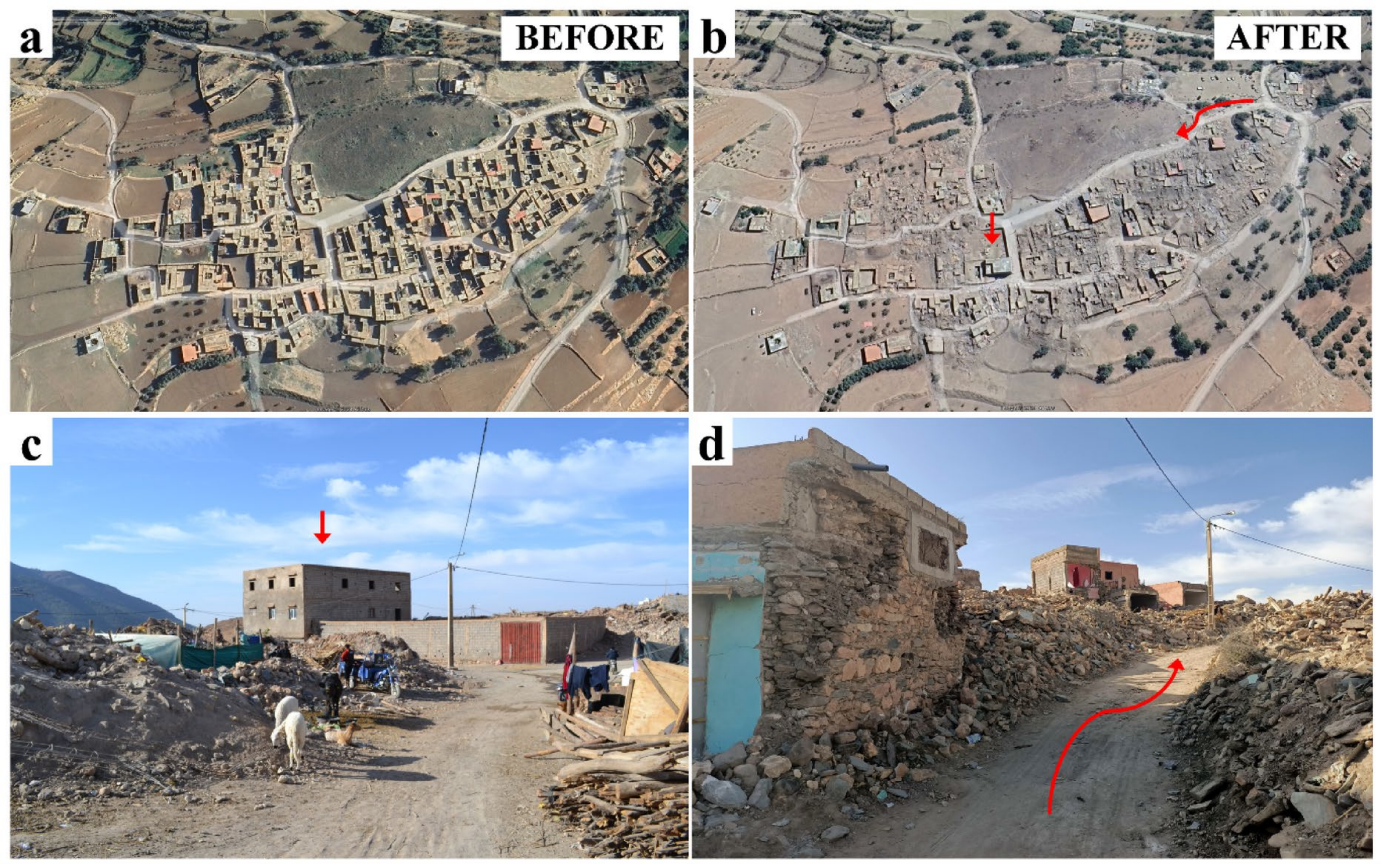
Google Earth images of the Tafeghaghte area (**a**) before and (**b**) after the Al Haouz event (GE Tafeghaghte^18,19^ Observed damage pointing (c) the location of the building and (**d**) the path indicated by red arrows in (**b**) (credit A. Imtiaz).

**Fig. 6:**
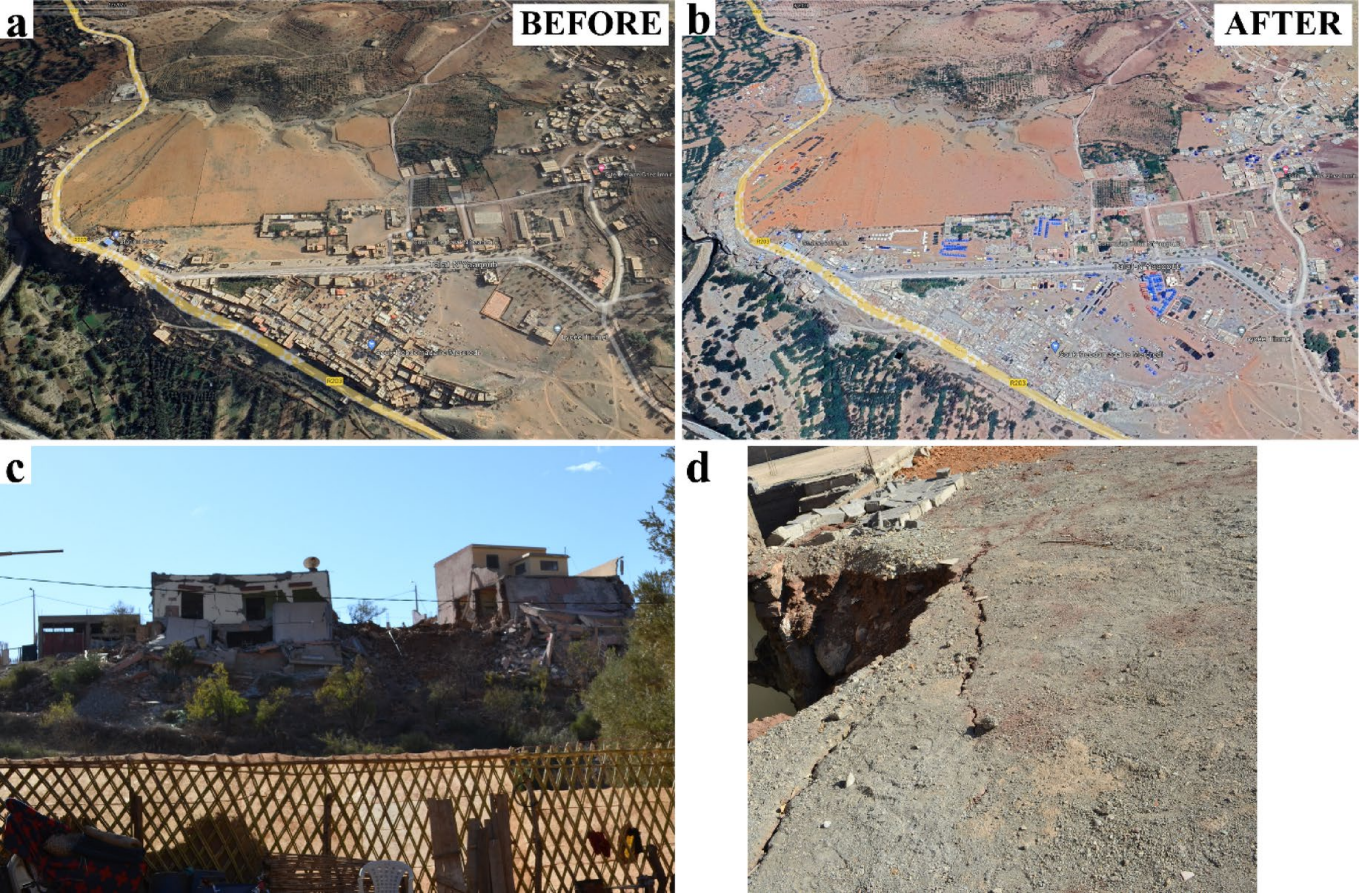
Google Earth images of the Talat N’Yaaqoub area (**a**) before and (**b**) after the Al Haouz event (GE Talat N’Yaaqoub^20,21^. (**c**) Observed damaged buildings on slopes, the location is marked in red rectangle on (**a**). (**d**) Observed ground failure (credit A. Imtiaz).

Figure 4 displays satellite images of Amizmiz, Tafeghaghte, Toufssirine, and Talat N’Yaaqoub areas, situated at the foothills and fringes of the mountains, where the most significant damage occurred. We can see that the Atlas Mountains exhibit a diverse topography, featuring high peaks, valleys, and plateaus. The topographic relief can focus and amplify seismic energy, particularly in narrow valleys or along the flanks of mountains. Irregularities in the topography, such as changes in slope, variations in elevation, and the presence of ridges or peaks, can focus and concentrate seismic energy. These geometric irregularities act as natural amplifiers, influencing the distribution of ground motion and creating localized amplification zones. Figure 5 demonstrates an overview of damage observed in the Tafeghaghte area (*R* = 15 km). This village was nearly leveled by the earthquake. Its location along with the extent and pattern of observed damage suggest a potential influence of resonance or complex site effects on seismic ground motion. Figure 6 (a, b) illustrates the before and after images of damage in Talat N’Yaaqoub area (*R* = 11 km). Numerous buildings in this area are constructed on slopes (e.g., Fig. 6c), revealing ground failures and lengthy cracks near the structures (Fig. 6d).

## Post-seismic management and social context

Post-seismic management requires significant resources and a preparedness phase. The acute response phase, including search and rescue, occurs immediately after the event. Initially, spontaneous community support is common, but it must be transitioned into organized, institutional efforts in the days and weeks that follow. Emergency assistance is needed, including healthcare, clean water, food, shelter, and hygiene items. Measures to secure buildings at risk of collapse must be implemented, and a safety assessment of damaged but standing structures is essential to ensure residents can safely return home. The response extends into the rehabilitation and reconstruction phases over the ensuing months and years.

The SGEB reconnaissance mission took place almost three months after the Al Haouz event, when the situation in the affected areas had significantly changed. The areas most severely impacted by the earthquake were situated along rugged, remote hillsides and valleys in Al Haouz province. Many of the mountain roads in the region were rendered impassable by boulders and debris from earthquake-triggered landslides. By the time of our visit, search and rescue operations had concluded, roads had been cleared to restore access, and humanitarian assistance was underway. Many emergency measures to secure affected buildings were observed in Marrakech (see Figs. 8, 9, 11, 13). While debris from collapsed and severely damaged buildings had been removed in some areas in Marrakech, this was not the case in the mountain villages. Tent camps were set up in most of the villages we visited to accommodate the affected populations. With the winter season approaching and nights being already cold by late November, especially in the mountainous areas, many people were awaiting the start of their reconstruction. Our interactions with residents in some areas, where buildings were visibly damaged but still standing, revealed that habitability assessments had been conducted by offices mandated by public authorities or possibly by volunteers. However, some people remained unsure about the habitability of their homes. Some families chose to use their damaged homes during the day and spend the nights in tents, demonstrating the level of risk accepted by individuals under constraints in such crises. We observed some recovery initiatives, in Amizmiz for example, where Mohammed VI Polytechnic University (UM6P) established temporary schools using modular structures as part of its ‘ReBuild’ program. In Talat N’Yaaqoub, we noted local rebuilding efforts using recycled debris from collapsed buildings.

## Damage in heritage and sacral structures

**Fig. 7:**
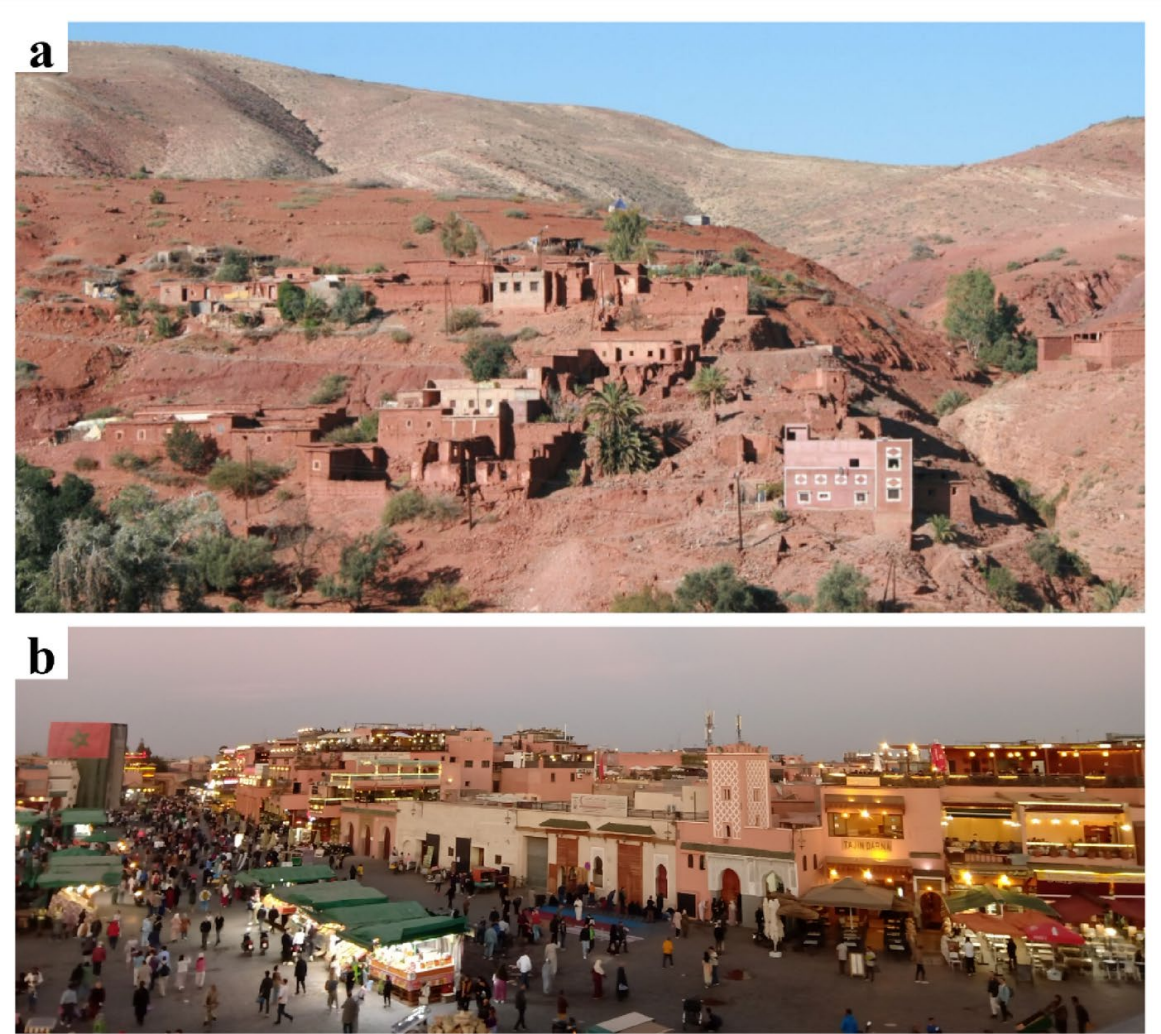
Typical buildings in (**a**) Toufssirine in the High Atlas (credit: S. Saloustros) and (**b**) in the old town Medina, Marrakech (credit: A. Imtiaz).

Morocco’s vast and culturally rich territory features diverse types of vernacular buildings that reflect local culture and environmental characteristics, varying significantly by location. For example, traditional constructions in the High Atlas (Fig. 7a) are markedly different from those in Marrakech (Fig. 7b). Despite its distance from the epicenter, the historic Medina of Marrakech, a UNESCO (United Nations Educational, Scientific and Cultural Organization) World Heritage site, suffered significant damage.

### Structural features of buildings in the Medina of Marrakech

The Medina of Marrakech exhibits a unique blend of traditional and contemporary architectural styles, evolved over time. Most structures within the Medina of Marrakech, whether residential or mixed residential-commercial, are constructed from adobe masonry, natural stone masonry, or a combination of both. Typically ranging from one to three floors, these buildings usually feature traditional, flexible timber floors. Within the densely packed old city, buildings are often juxtaposed forming aggregates. Their seismic vulnerability depends not only on their own structural and material characteristics but also on their position within the aggregate, the geometric and structural features of adjacent buildings, and the connections between them^22^. Additionally, this vulnerability could be exacerbated by a lack of maintenance and unregulated modifications, including extensions that negatively impact structural behavior.

**Fig. 8:**
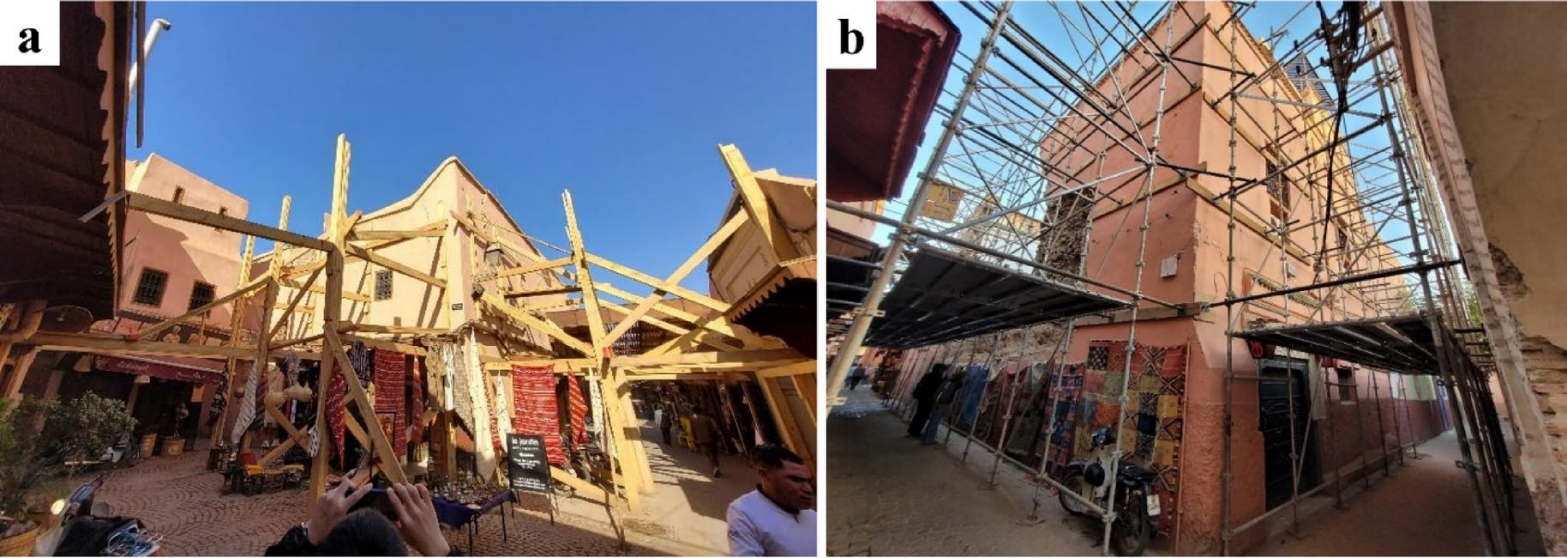
Examples of propping and shoring observed in in the old town Medina, Marrakech. (**a**) Opposite wooden shoring, and (**b**) facing steel supports for a corner building (credit E. Lattion).

**Fig. 9:**
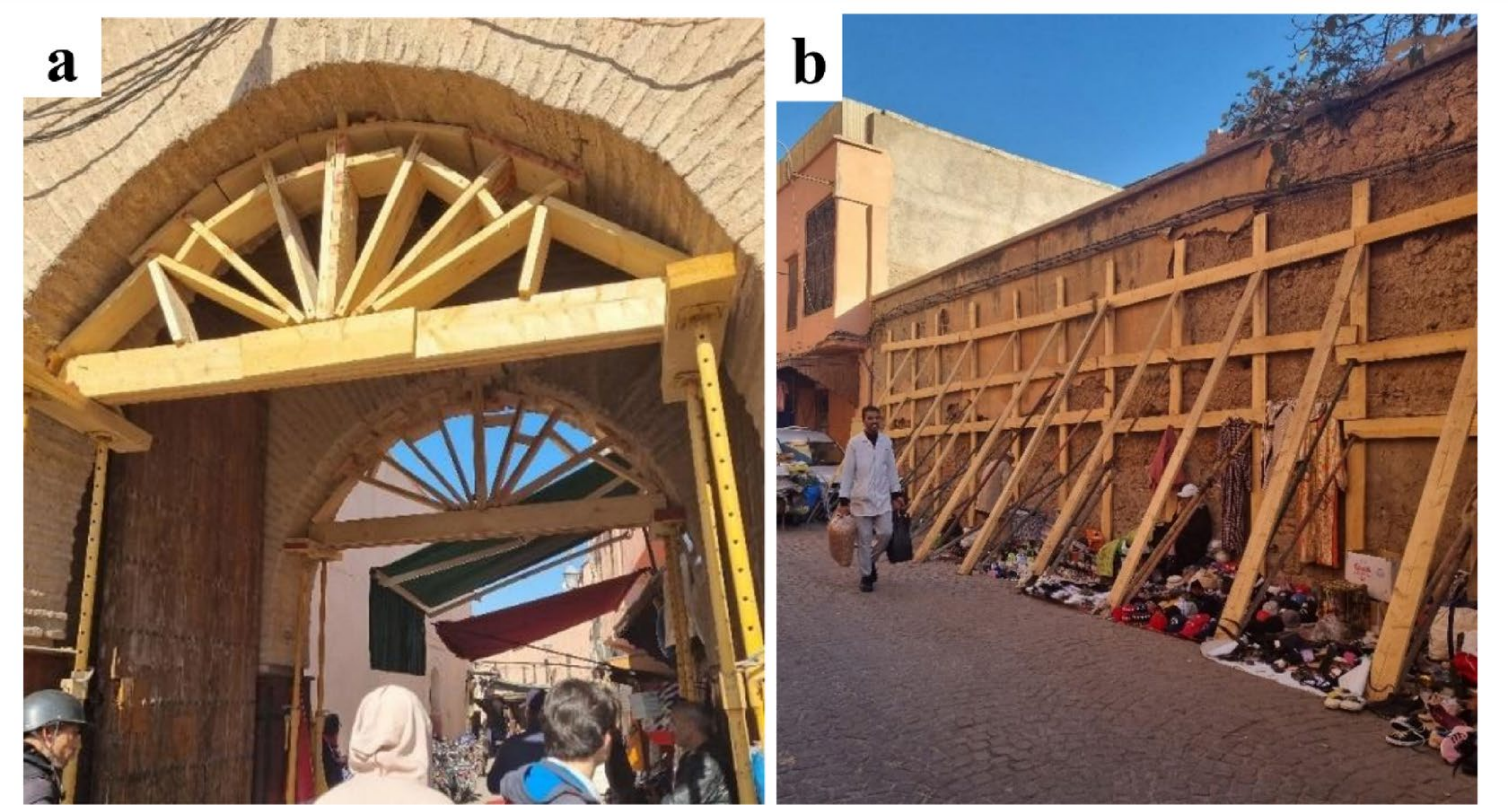
Examples of propping and shoring observed in in the old town Medina, Marrakech. Propping with (**a**) bending wooden arches for the arched beams, and (**b**) crutch support for a section of the outer wall (credit G. Cortés).

### Damage in the Medina of Marrakech

A common damage typology within Medina was the overturning and collapse of façade walls, as demonstrated by the widespread use of shoring to prevent out-of-plane deformation, especially on corner buildings where damage was concentrated (Figs. 8 and 9).

**Fig. 10:**
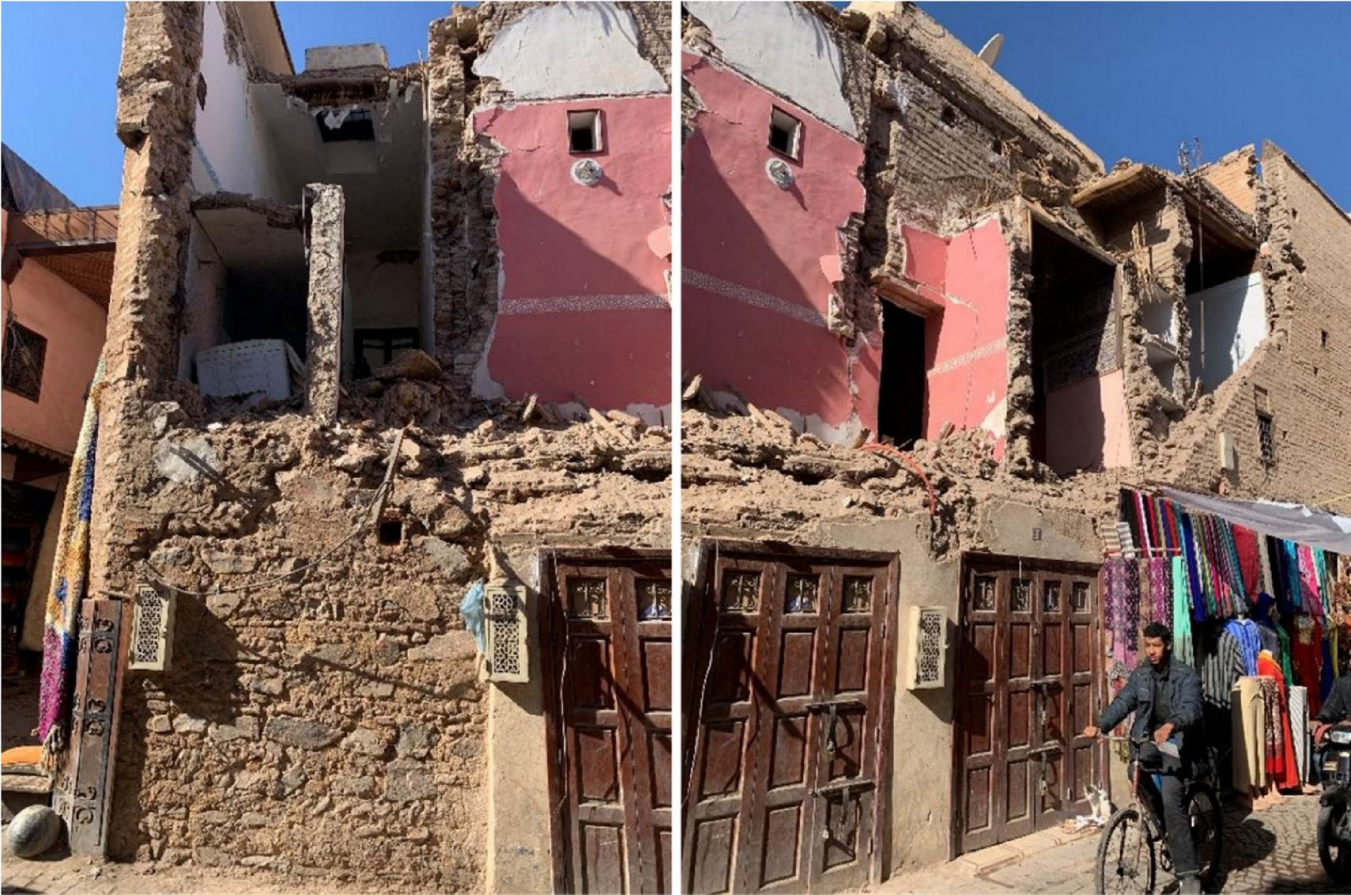
Out-of-plane collapse of a building façade in the Medina of Marrakech (credit M. Devaux).

Façade overturning collapse is usually the result of a combination of factors, such as the existence of weak interface zones between different materials (i.e. natural stone and adobe masonry), the insufficient interlocking of stone masonry at the corners or the low strength of the material resulting in crack opening, the presence of flexible floors that do not allow a redistribution of forces and thus a box behavior, and the geometrical irregularity of the interior of the structure resulting in an irregular force distribution. Figure 10 presents an example of a façade overturning in the Medina of Marrakech featuring most of the aforementioned structural vulnerabilities.

### Damage to mosque structures

**Fig. 11:**
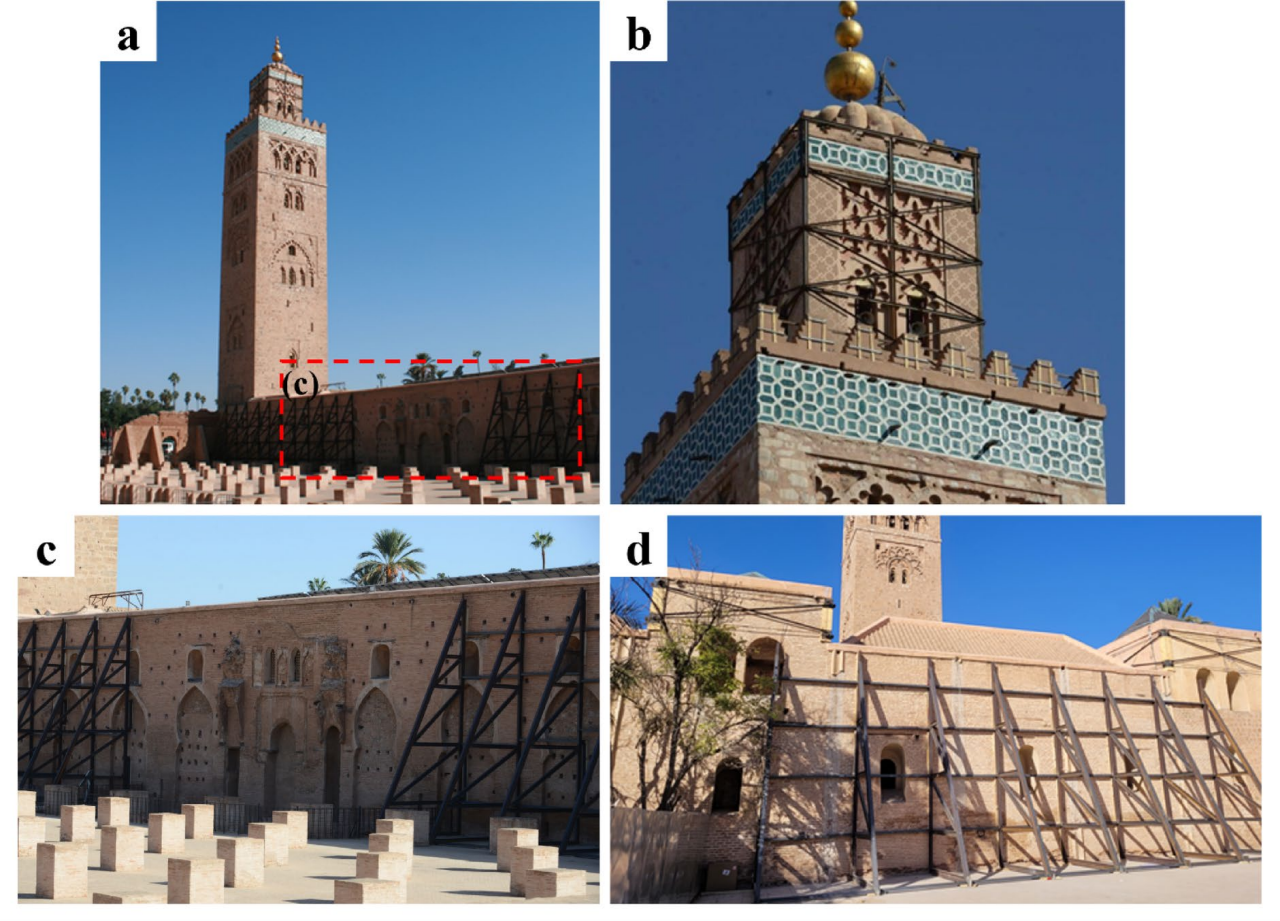
Observations at the Koutoubia Mosque, Marrakech. (**a**) View of the northwest façade and minaret (credit M. Devaux). Stabilization measures at the (**b**) top of the minaret (credit Y. Zhu), and (**c**,**d**) perimeter walls (credit Y. Zhu and E. Lattion).

**Fig. 12:**
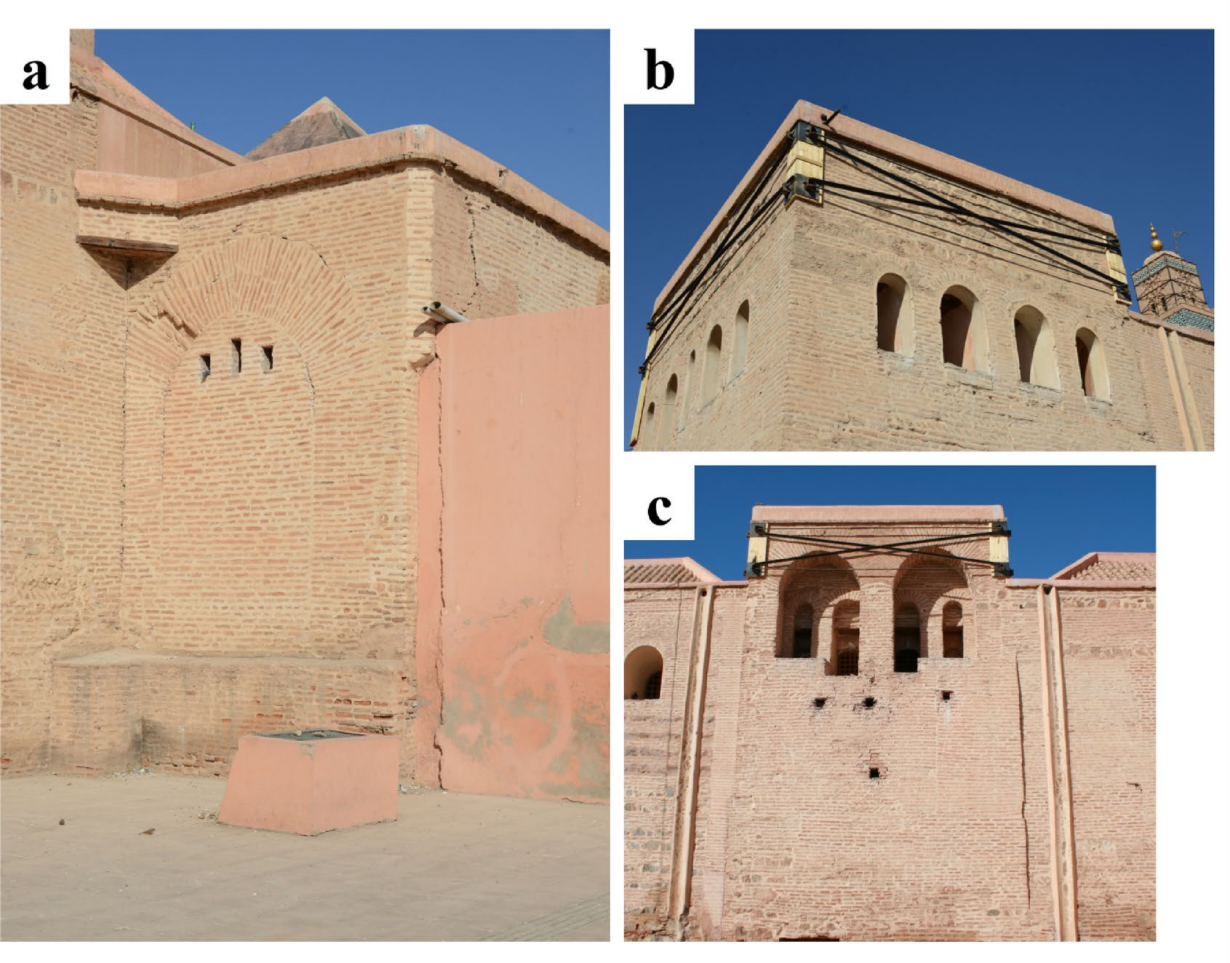
(**a**) Damage near the ‘minbar’ along the ‘Qibla’ wall, (**b**,**c**) stabilization measures at the top of extruding structures in the perimeter of the Koutoubia Mosque (credit S. Saloustros).

**Fig. 13:**
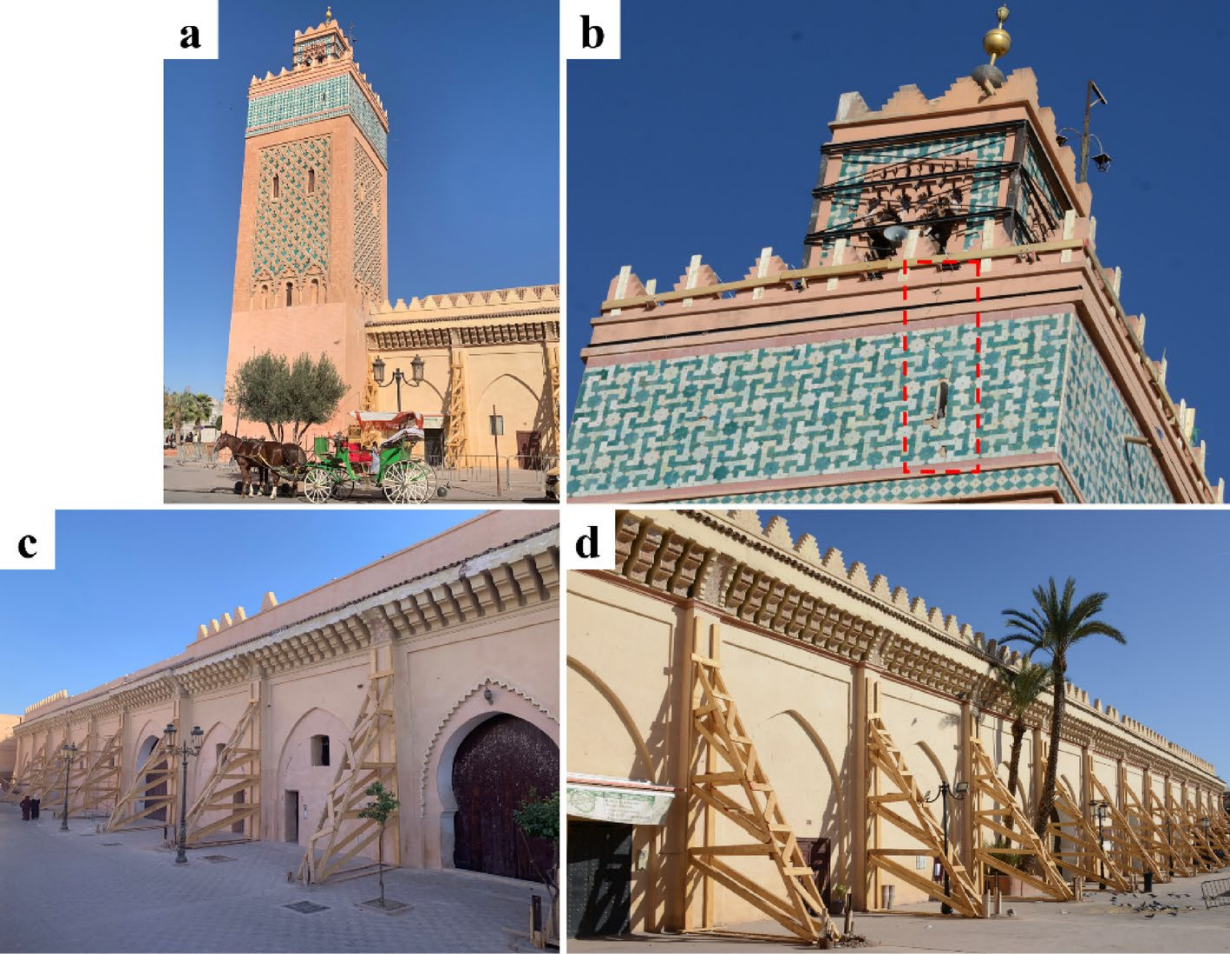
Observed damage and emergency interventions for safety measures at the Kasbah Mosque, Marrakech. (**a**) View of the minaret (credit M. Devaux), (**b**) cracking and stabilization measures at the top part of the minaret (credit Y. Zhu), (**c**) cracking between the intrados of arches and the later built masonry walls (credit Y. Zhu), (**d**) stabilizing measures with timber buttresses at the perimeter walls of the mosque (credit M. Devaux).

Mosques exhibit architectural features and structural elements such as arches, domes, vaults, towers, minarets, arcades, and buttresses. This structural complexity usually makes them highly vulnerable to earthquakes. To illustrate this point, we address the cases of two most significant historic mosques in Marrakech, Koutoubia (Figs. 11 and 12) and the Kasbah (Fig. 13), which sustained major damage but did not collapse. Due to access restrictions, our observations are limited to externally visible parts.

The Koutoubia Mosque, located in the southwest Medina quarter, is the largest mosque in the city. This 12th-century structure is approximately 90 m (m) wide. It is 57 m and 66 m long on its west and east sides, respectively^23^. It was primarily built of brick, with sandstone masonry used for parts of the outer walls. The mosque’s 77 m tall minaret tower is square-shaped and constructed with rubble masonry using sandstone. It consists of a square shaft measuring about 13 m per side for roughly four-fifths of its height, above which a smaller shaft measuring about 7 m per side is topped by a finial with three metal spheres. The height-to-width ratio of the minaret is slightly over 5-to-1, making it taller and slenderer compared to contemporary mosques.

During the earthquake, the minaret was seen shaking back and forth alarmingly, emitting clouds of dust, and raising fears of collapse^24^. While it withstood the quake, the minaret of Koutoubia was reported to sustain large cracks (UNESCO news 2023). During our visit, we observed cracks on the upper part of the minaret (Fig. 11). The spire was stabilized using a steel bracing around its perimeter and across its height (Fig. 11b), aimed to prevent any further damage in case of future earthquakes by allowing the transfer of forces from out-of-plane loaded walls to the perpendicular walls, which have higher in-plane stiffness. Steel and timber bracing elements were also used to stabilize the parapets in the balcony against out-of-plane overturning. Apart from the tower, buttressing elements from steel were placed to stabilize the perimeter walls of the mosque in several locations, as can be seen in Fig. 11 (c, d) at the lateral walls.

Figure 12 (a) shows the out-of-plane failure at a corner of an extruding part along the perimeter of the mosque, which includes vertical cracking between orthogonal walls and cracking at the interface between the extrados of the arch and spandrel atop. Cracking and out-of-plane deformation are also visible to the lower left part of the arch, which probably served in the past as an entrance. Stabilizing steel bracing was commonly found around vertically extruding parts of the structure, which probably experienced cracking due to the lack of lateral constraints (see Fig. 12b, c).

The Kasbah Mosque, also built in the 12th century, is located in the southern part of the old Medina. The mosque is roughly square in plan and primarily constructed from brick masonry, with wooden elements in the ceilings. The minaret, like that of the Koutoubia Mosque, has a square base measuring about 9 m per side. Its plain walls are built of rubble stone up to the roofline, with the upper portion made of brick. Atop the base is a smaller secondary shaft measuring about 4 m per side, surmounted by the spire^25^.

Similar to the Koutoubia minaret, observed external cracks at the upper minaret as well as below the balcony (Fig. 13a, b). Steel bracing was already in place to stabilize the top part of the structure. Separation cracks could be seen between the intrados of arches and later-constructed masonry walls in the perimeter walls of the mosque (Fig. 13c). Provisional timber buttresses were placed to stabilize and prevent out-of-plane overturning of the perimeter walls (Fig. 13d), which may have experienced increasing horizontal thrust from the vaults and arches within the mosque during the seismic action.

**Fig. 14:**
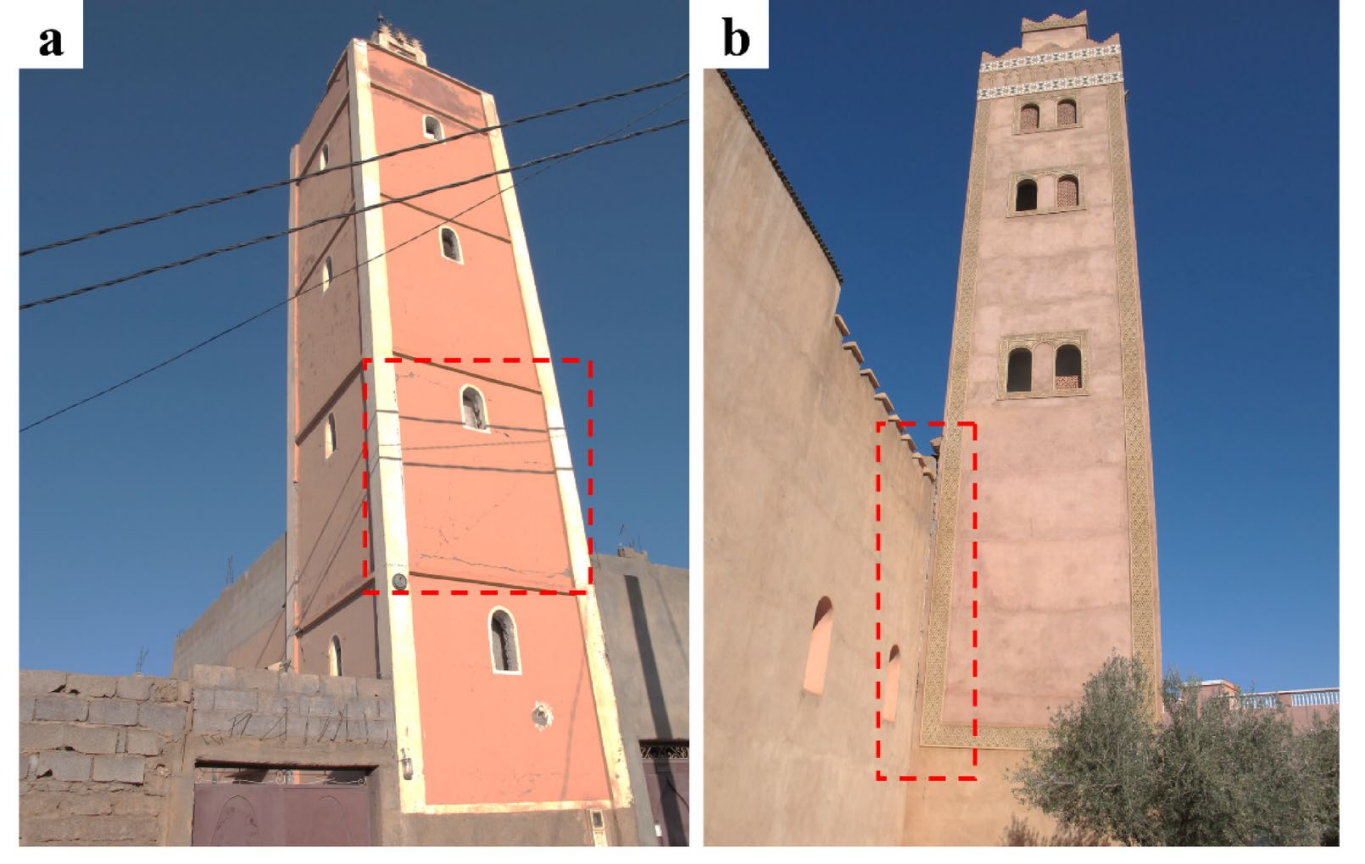
Observed cracks (**a**) on the minaret of a mosque in Toufssirine with and (**b**) at the connection between the minaret and the mosque structure in Ouirgane (credit M. Devaux).

It is important to note that minarets, due to their slender structure, have a relatively longer fundamental resonance period compared to the adjacent mosque structure. As a result, it is common to see cracks at the interface between the minaret and the mosque (Fig. 14). Vertical cracks can often be observed along this interface, while sub-horizontal and oblique cracks are typically found at the height of the mosque’s roof, as we observed in several mosques. We observed similar damage patterns in relatively recent RC mosques (Fig. 14).

## Contemporary buildings in the urban areas

**Fig. 15:**
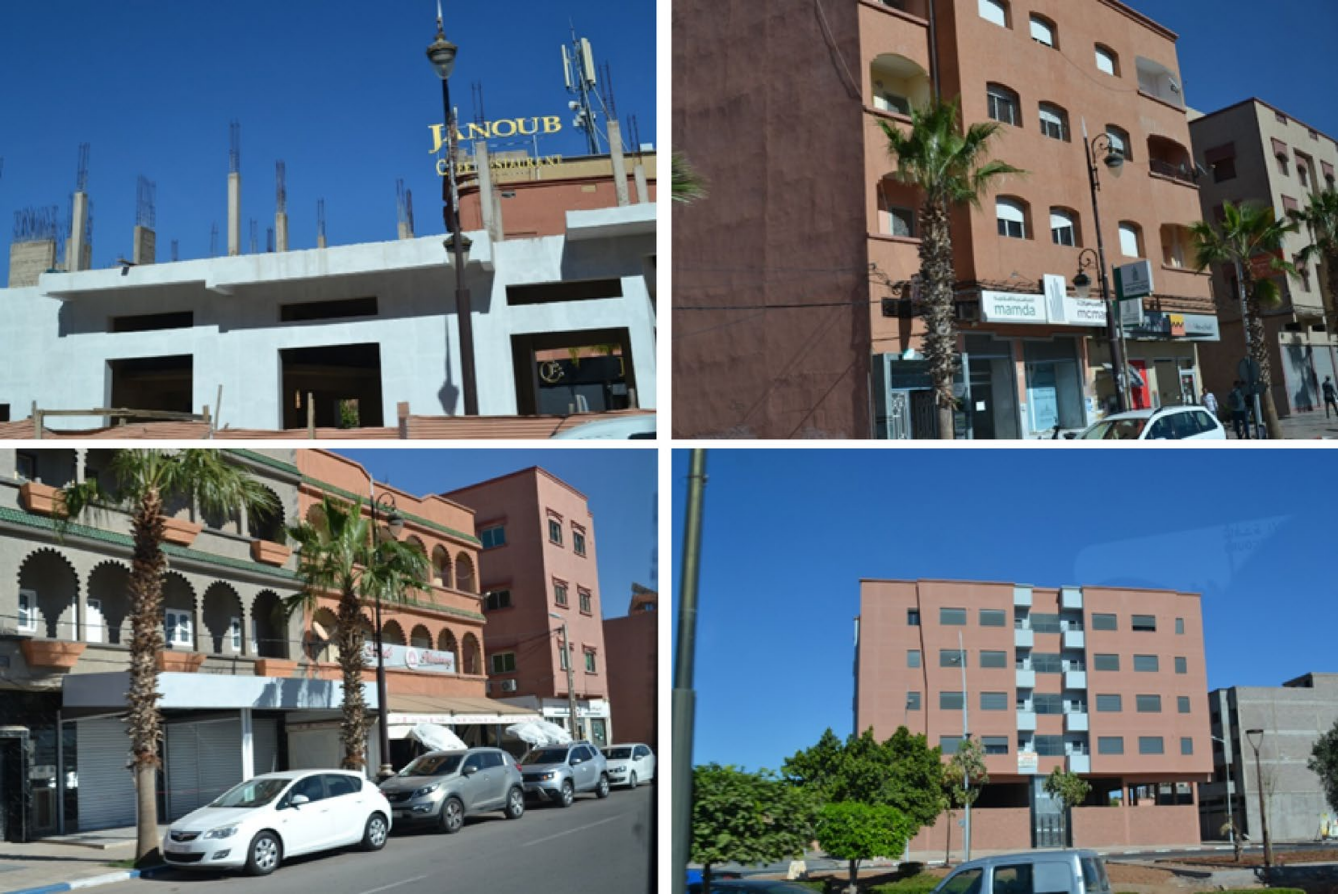
Examples of some contemporary buildings in and near Marrakech (credit A. Imtiaz).

Contemporary buildings in Marrakech generally fared better than traditional structures, as they were more likely to incorporate earthquake-resistant design elements. Historically, Moroccan architecture has been characterized by features such as intricate geometric patterns, colorful tilework, and ornate arches. In modern constructions, these aesthetic elements are often integrated with improved engineering practices to ensure structural integrity (Fig. 15).​ Contemporary urban buildings in Morocco, particularly in seismic regions like Marrakech, are predominantly composed of reinforced concrete (RC) frames with unreinforced masonry infill walls often made of hollow concrete blocks. This RC construction typology dominates the building stock^26,27^ constructed after the adoption of the Moroccan seismic code RPS2000 (updated in 2011). In accordance with its requirements, recently constructed buildings can also be designed with earthquake-resistant shear walls, which has been confirmed by observations of buildings under construction. As a result, the code-compliant RC buildings in Marrakech are more likely to exhibit improved seismic behavior compared to older, non-engineered structures, which often lacked proper reinforcement and lateral load-resisting systems.

**Fig. 16:**
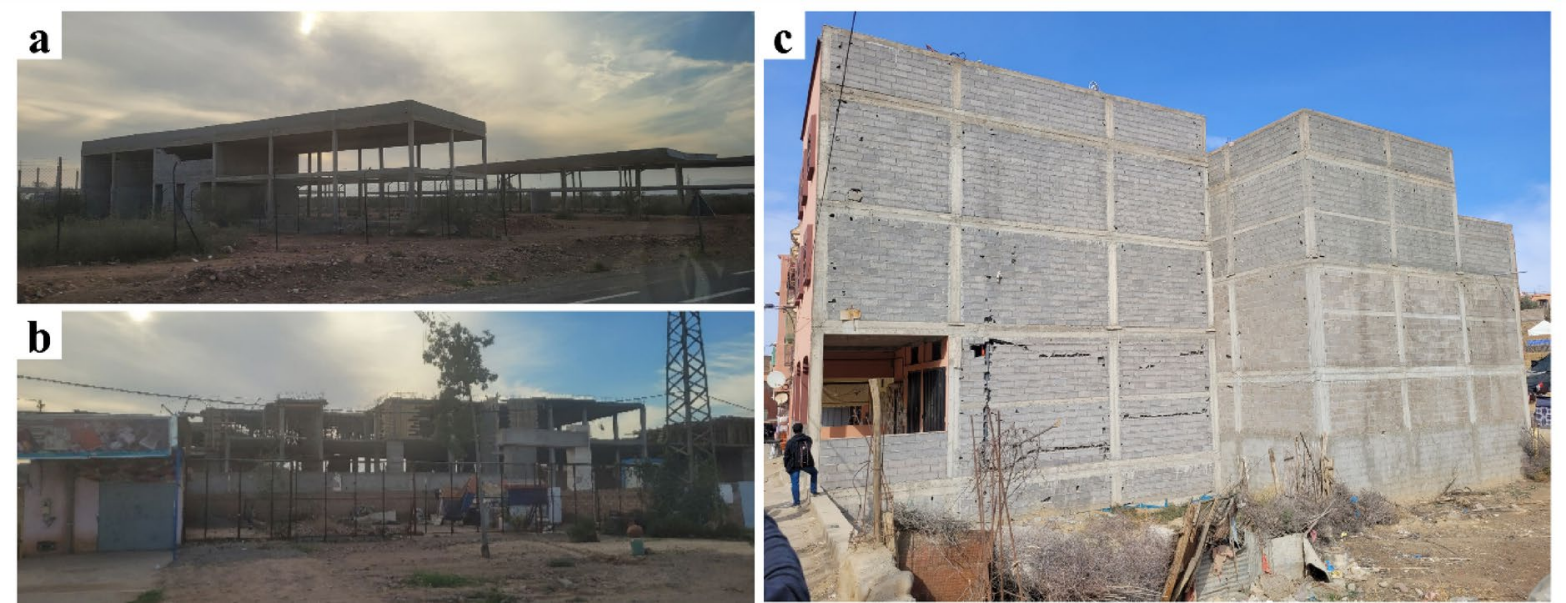
(**a**) reinforced concrete frame-type buildings under construction, and (**b**) reinforced concrete frame-type buildings under construction with reinforced concrete shear walls near Marrakech (credit: E. Lattion). Add description for (**c**) reinforced concrete frame structure with minor damage at the masonry infill walls in Amizmiz.

In the areas near Marrakech, the buildings under construction we observed were either “frame-type” structures, with reinforced concrete frames providing lateral resistance and infill made of non-load-bearing brick masonry (Fig. 16a), or “shear wall-type” structures with reinforced concrete shear walls, with or without accompanying frames (Fig. 16b). These building typologies, located relatively far from the epicenter (80 km), were not inspected and are therefore not included in the scope of this report. Figure 16c shows an example of reinforced concrete frame structure in Amizmiz. Buildings of this typology did not exhibit significant structural damage externally, although minor non-structural damage was observed in the infill masonry walls.

## Reinforced concrete-masonry hybrid buildings

### Structural characteristics

**Fig. 17:**
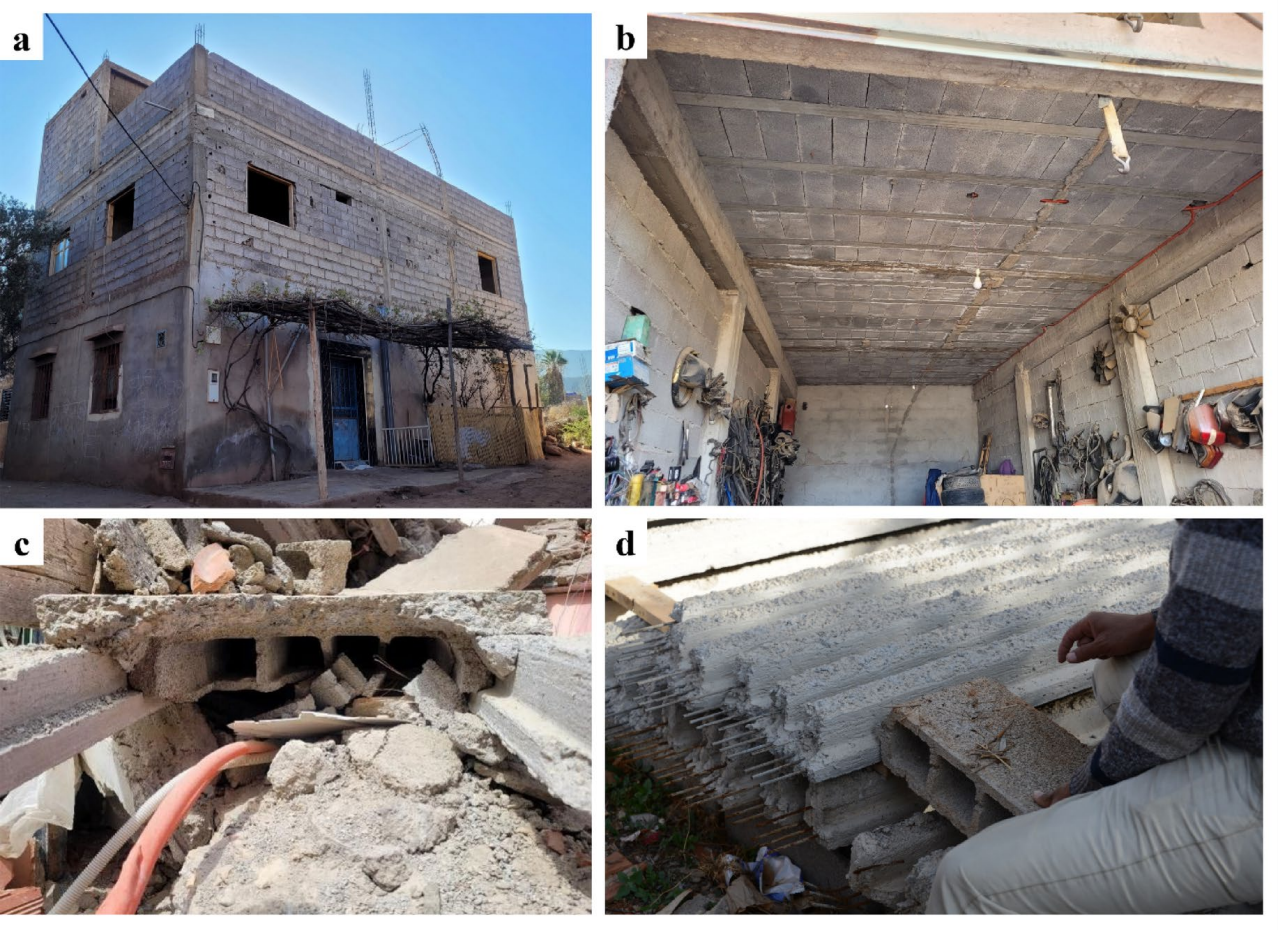
(**a**) A typical residential building in the High Atlas, with reinforced concrete pillars and beams, masonry infill, expanded over several years or even generations. (**b**) Typical beam-block floor typology in a building in Amizmiz, (**c**) close-up view of a collapsed floor in Amizmiz (credit E. Lattion), and (**d**) precast beams during the visit to an artisanal brick factory close to Amizmiz (credit Y. Zhu).

Most of the severe building collapses were concentrated in older buildings and in rural villages in the Atlas Mountains region closer to the epicenter, rather than in modern constructions in Marrakech. In these regions, reinforced concrete and modern concrete block masonry are two materials widely used for the construction of residential and public buildings (Fig. 17a). Many of these buildings were constructed before the publication of the Moroccan code RPS2000. The phased construction of these buildings is evidenced by the presence of exposed reinforcement corresponding to a probable future vertical extension at the roof levels of several buildings. The structural characteristics of many of the surveyed reinforced concrete and modern masonry buildings in the rural areas show significant variation, as it depends on local construction know-how, as well as the quality, availability, and access to construction materials.

**Fig. 18:**
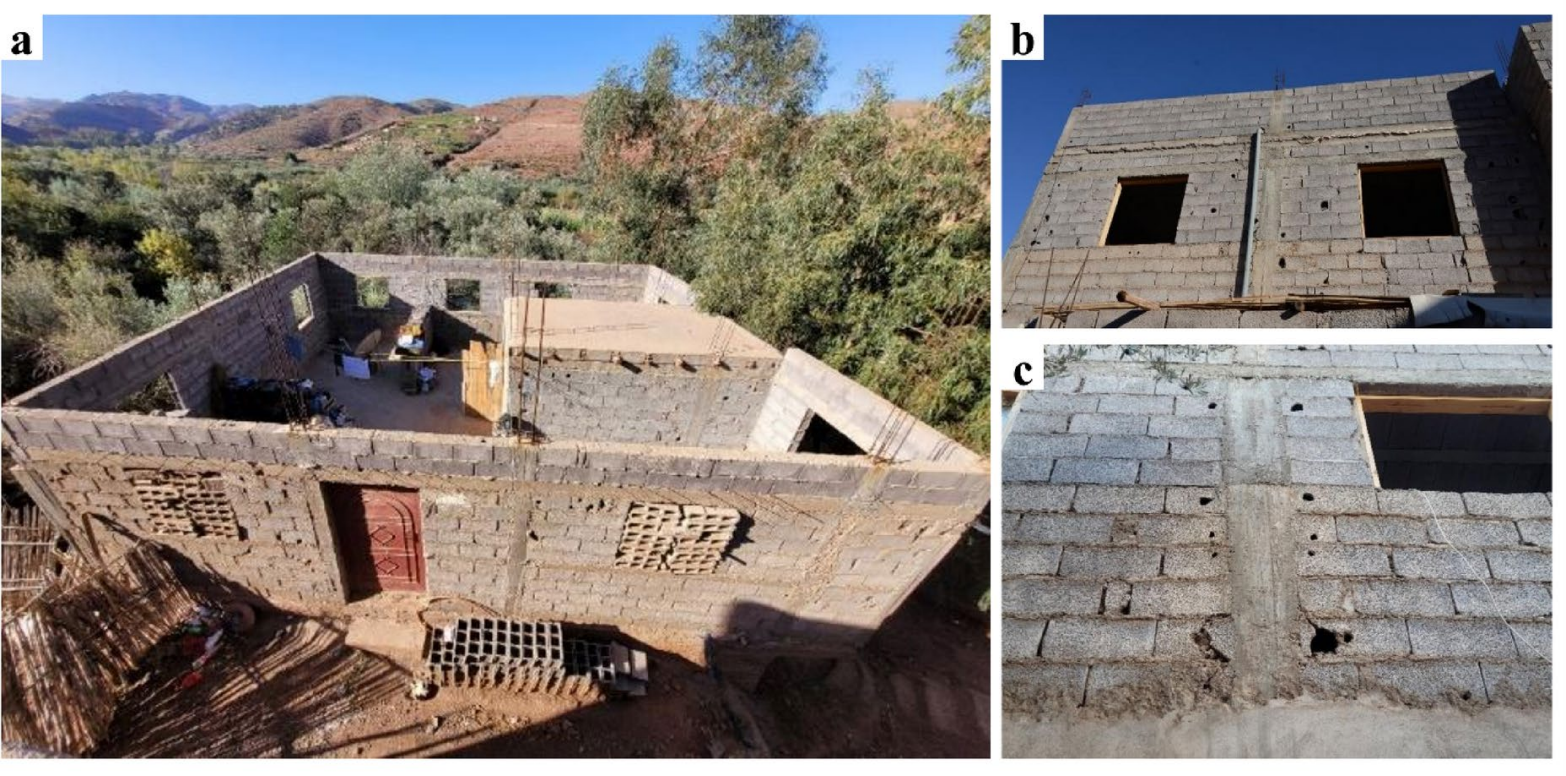
(**a**) A building of hybrid structural typology, with reinforced concrete and concrete masonry blocks, under construction in Toufsirrine, and (**b**,**c**) close-up view of the connection between pillars and walls, also showing the low quality of the masonry blocks (credit E. Lattion).

As shown in Fig. 17 (b), the slabs are generally made of precast reinforced concrete beams with masonry infill. An additional site-cast unreinforced concrete cover with a width between 40 and 80 mm forms the top layer. The top surface of the precast beams presents a high roughness to improve the friction connection between them and the concrete cover (Fig. 17c, d). In many buildings, the reinforced concrete pillars and ring beams connecting the floor with the walls were cast after the construction of the masonry walls. The inspection of buildings with floor or wall collapse indicated that there was no mechanical connection between the masonry walls and the perimeter beam (ring beam) to the floor slab. The reinforcement ratio and the dimensions of the reinforced concrete elements appear, in most cases, to be lower than those of a reinforced concrete frame structural system (Fig. 18). Additionally, the construction details in the connection between reinforced concrete elements and masonry walls, as well as insufficient reinforced concrete elements around openings, do not correspond to the seismic design of a confined masonry structural system (Fig. 18a–c). Therefore, this construction typology is considered as a hybrid between reinforced concrete and confined masonry and will be referred to hereafter as hybrid RC-masonry.

**Fig. 19:**
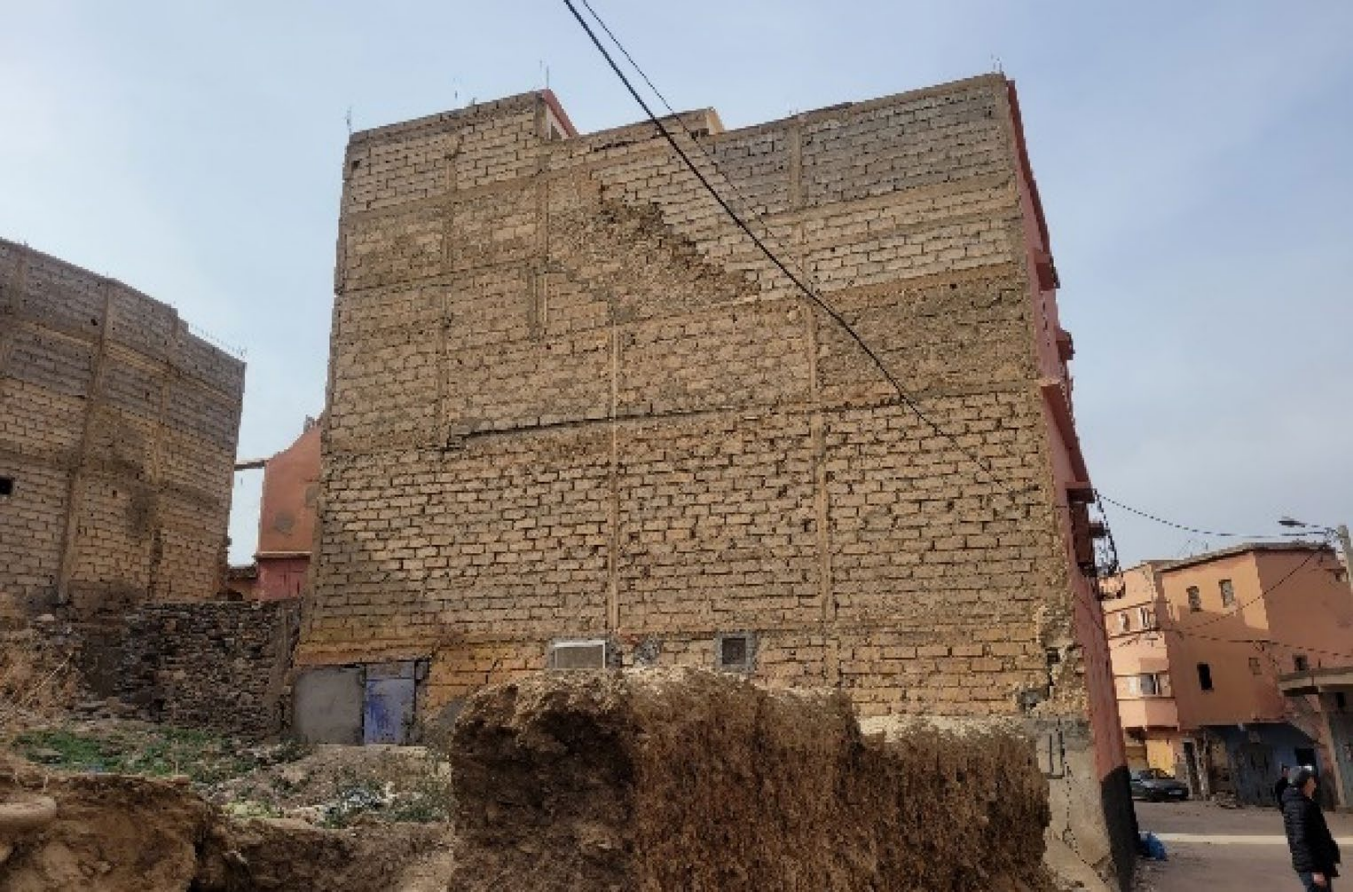
A hybrid RC-masonry building in Amizmiz, where beams and columns are arranged irregularly (credit E. Lattion).

It is to be noted that the Moroccan code RPS2000 does not treat confined masonry as a seismic bracing system, and therefore these empirical constructions present significant variability in typologies and material quality. This variability makes it difficult to define general building dimensions and material properties observed in the inspected structures. The distinction between frame and hybrid RC-masonry structures is not always obvious. In towns like Amizmiz, buildings tend to be taller and seem to be constructed as reinforced concrete frame systems with masonry infills. As shown in Fig. 19, this is not always the case, because of the lack of regularity in the horizontal and vertical arrangement of beams and pillars.

### Damage in hybrid RC-Masonry buildings

Hybrid RC-masonry buildings further away from the epicenter, as for instance in Toufsirrine, did not exhibit significant damage leading to collapse. On the other hand, in the rest of the visited areas closer to the epicenter, we could observe important damage and collapses of these structural typologies. In Talat N’Yaaqoub, one of the most affected municipalities 11 km away from the epicenter, we could closely inspect two buildings within this category with significant damage but no complete collapse. The two buildings appeared to be relatively new and were situated on a hillside with earthen embankment on one level at the rear side.

**Fig. 20:**
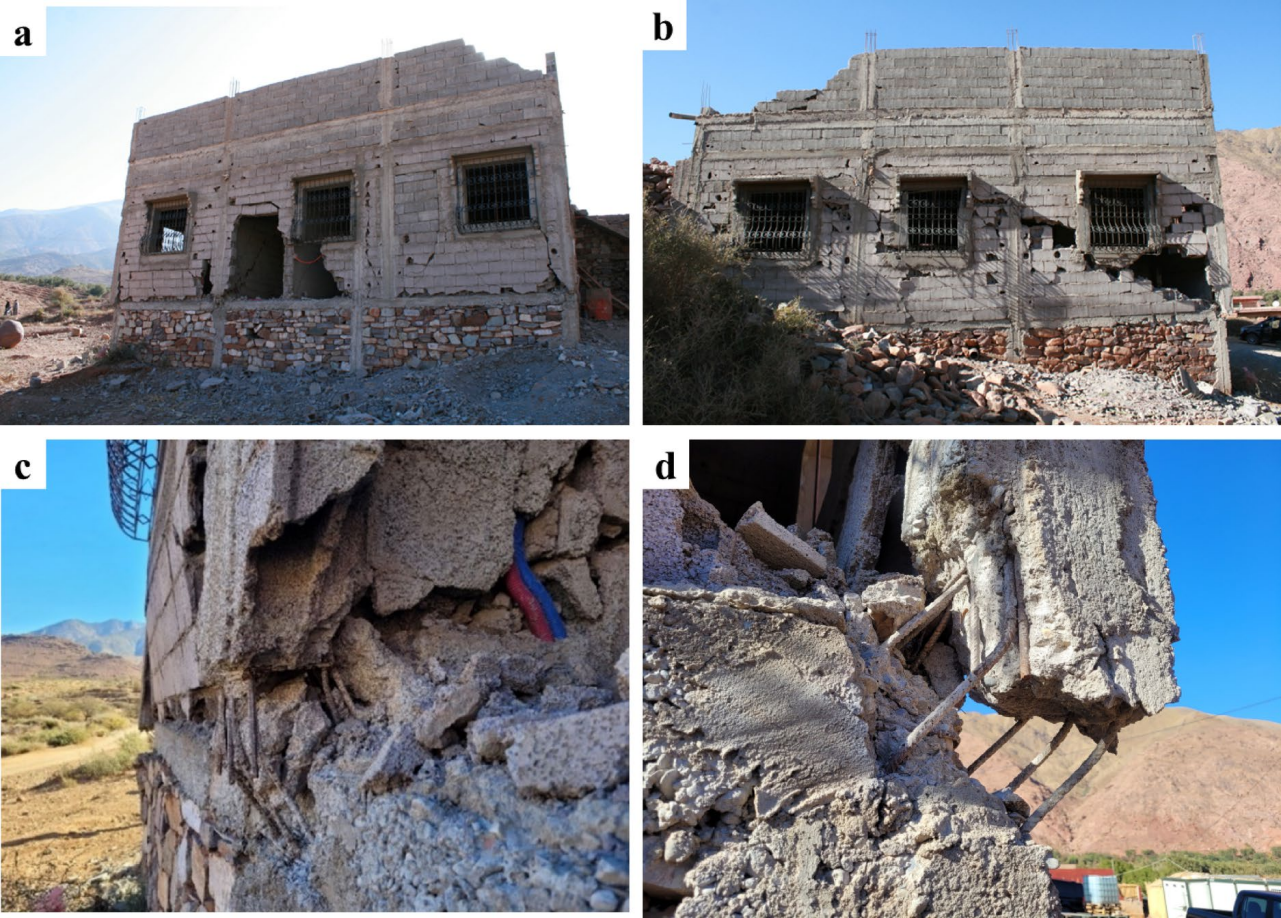
Damaged (**a**) front and (**b**) lateral façades of a hybrid RC-masonry structure in Talat N’Yaaqoub (credit S. Saloustros). Close-up of shear failure of the reinforced concrete pillars in the (**c**) middle and (**d**) corner of the façade (credit E. Lattion).

The first building, presented in Fig. 20 (a), was constructed using the presented hybrid RC-masonry system with floors made from the beam-block structural typology. At the ground-level, the masonry walls are made from stone masonry instead of concrete blocks. Discussions with locals revealed that this is a common practice aimed at reducing construction costs associated with modern materials. On top floors, the masonry walls were made of concrete blocks. These blocks are constructed in local facilities that we had the opportunity to visit and are characterized with low strength and high fragility. Reinforced concrete elements were casted after the construction of the walls and the concrete appears to be of poor quality and insufficiently vibrated, manifested by the non-homogeneous distribution of aggregates across the section. An irregularity in the vertical distribution of the piers on the front façade is also visible (Fig. 20a). The masonry walls on the two lateral façades presented important diagonal shear cracking due to the formed compression strut (Fig. 20b). This compression strut led to shear failure at the bottom of the concrete pillars (Fig. 20c, d). A close look at the reinforcement at the base of the piers manifests the lack of stirrups and the presence of lap splices, which increased the vulnerability of the pillars against a brittle shear failure. Overall, the significant damage in the structure is due to the combination of structural and material vulnerabilities such as the absence of confining elements around the openings, the insufficient connection between reinforced concrete and masonry elements, the low quality of the concrete and masonry units, and the low reinforcement ratios.

**Fig. 21:**
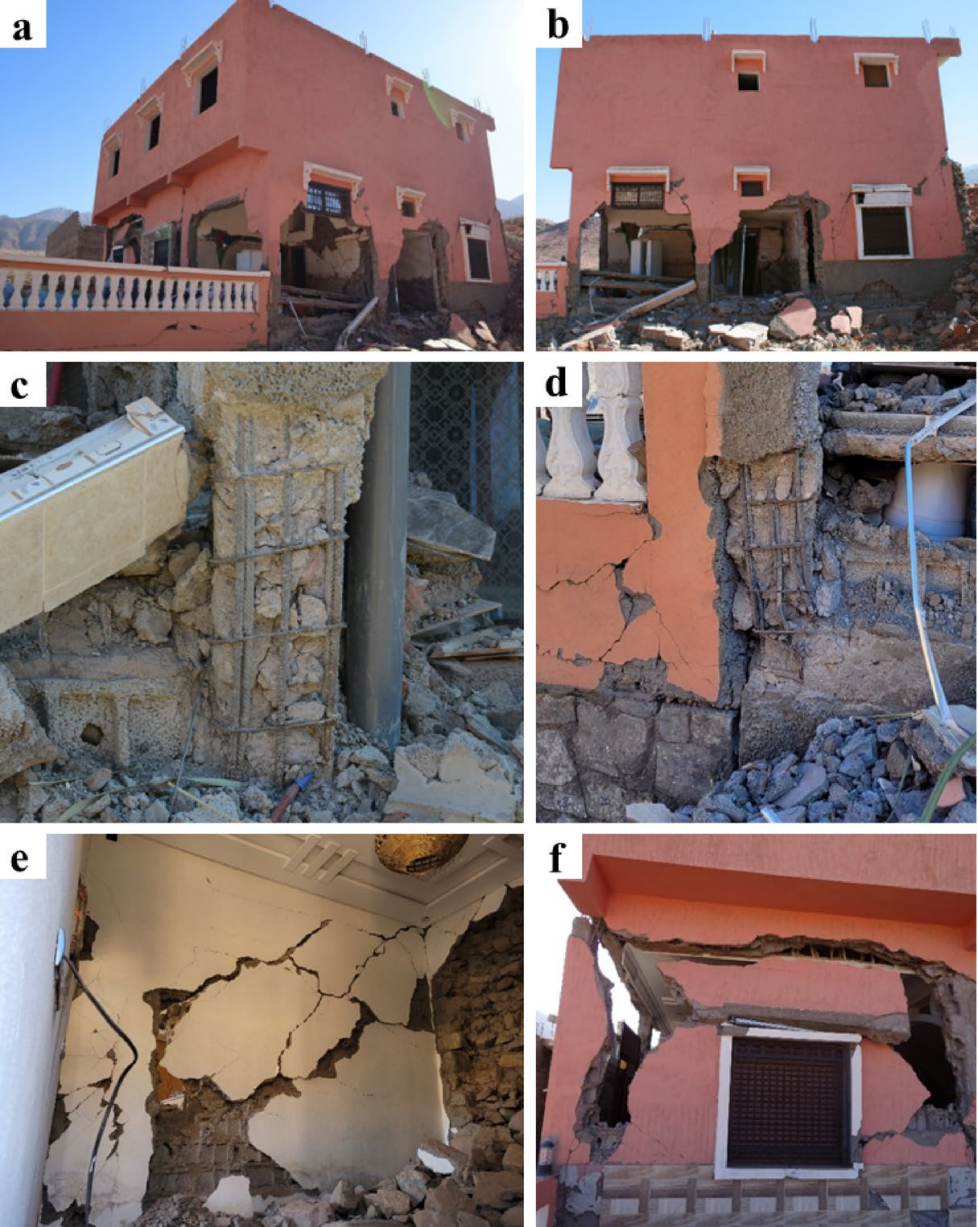
(**a**) Corner and (**b**) lateral view of a damaged hybrid RC-masonry structure in Talat N’Yaaqoub (credit M. Beqiraj and S. Saloustros). (**c**,**d**) Close-up of the damage at the base of the ground-floor piers (credit Y. Zhu and E. Lattion), (e) shear cracking in masonry walls (credit E. Lattion), and (f) damage in the wall and column at the front façade (credit H. Sehaqui).

We present the second building, located adjacent to the first one, in Fig. 21. Compared to the former one, it has an additional floor level and, in general, it presents a better quality of materials and construction details. In this case, the reinforcement ratios are higher at the piers, as seen in Fig. 21 (c, d). Like the former one, lap splices are also present at the base of the piers, reducing the ductility of these elements.

Masonry walls cracking in the front and lateral façade (Fig. 21a, b) collapsed or were heavily damaged due to shear failure (Fig. 21e). Those at the front façade exhibited an out-of-plane collapse due to lack of axial force and connection with the reinforced concrete elements. The reinforced concrete piers presented important damage forming hinges at their two ends due to shear cracking (see Fig. 21c, d, f). Note the absence of stirrups at the top of the piers (Fig. 21f). The failure of the concrete (see Fig. 21c, d) shows that its quality and strength were insufficient. The large distance between stirrups led in some cases to the buckling of the longitudinal reinforcement (Fig. 21d).

For both buildings (Figs. 20 and 21), the horizontal forces, and deformations due to the seismic actions were accentuated by the thrust of the embankment located upstream. The cracks of several centimeters (example shown in Fig. 6c, d) were visible on the ground just 2 m away from the building (Fig. 20), which show decompression of the ground and confirm the downward movement of the building.

**Fig. 22:**
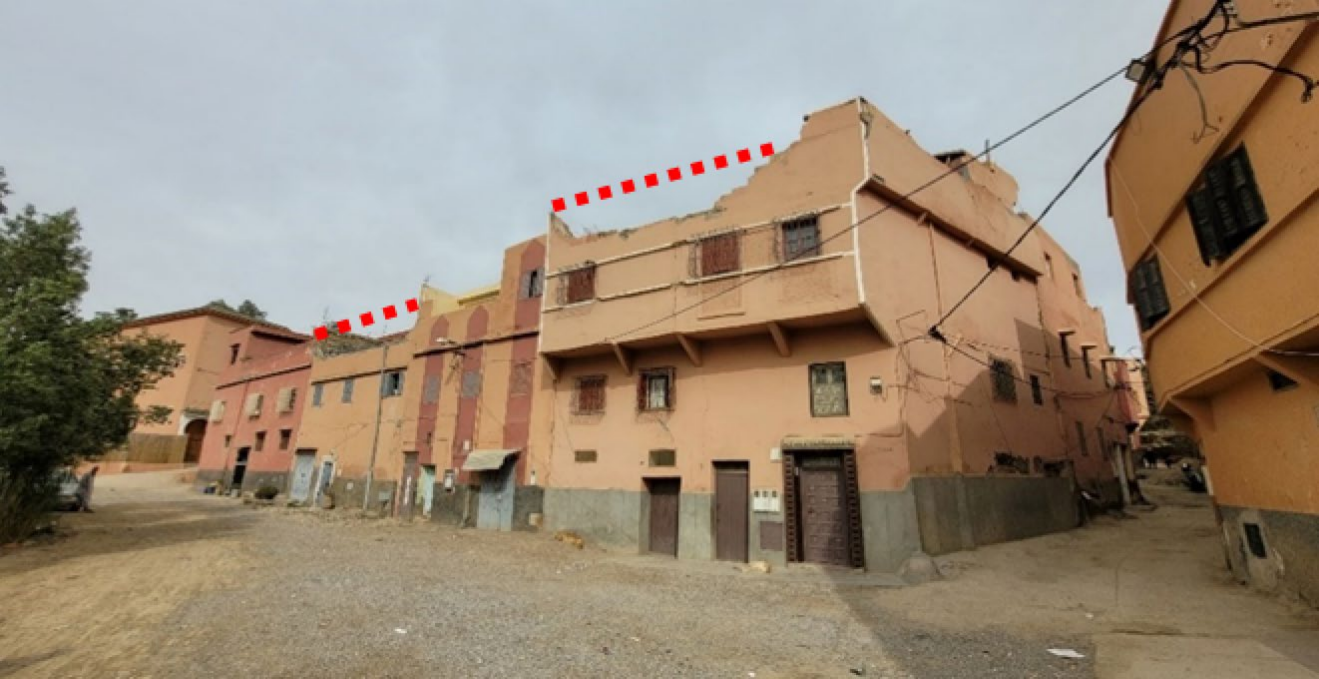
Aggregate hybrid buildings in Amizmiz showing locations with out-of-plane failure of parapets (credit E. Lattion).

**Fig. 23:**
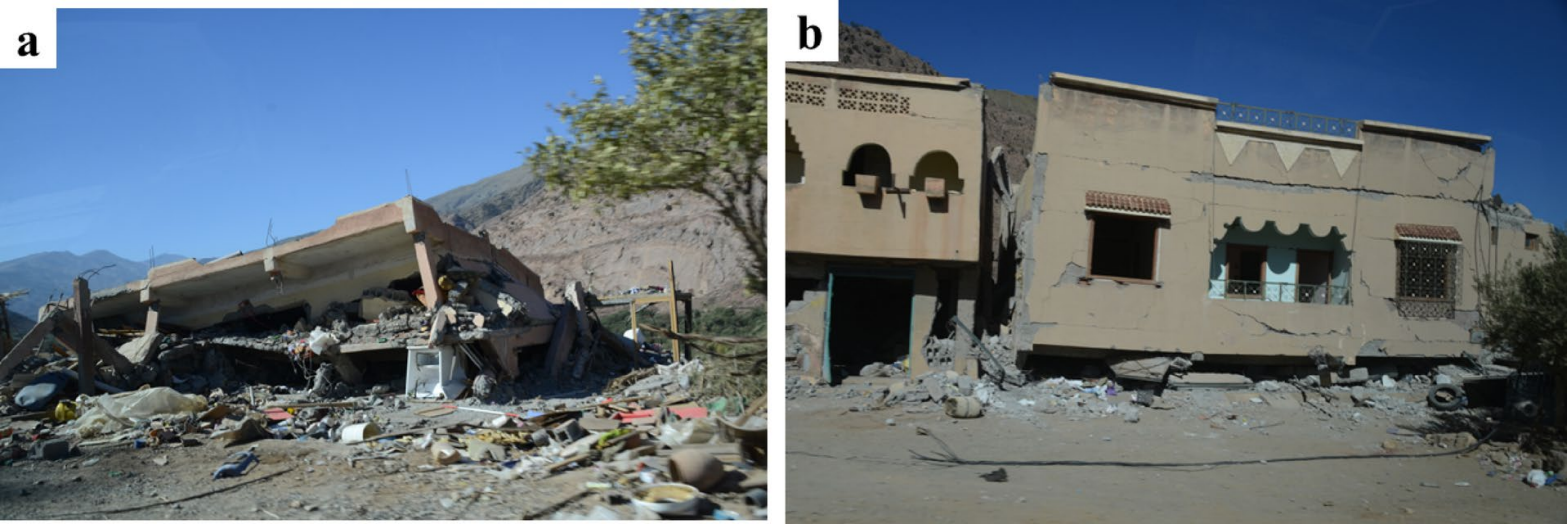
(**a**) Collapsed reinforced concrete frame building, and (**b**) soft-story collapse in a building in Talat N’Yaaqoub (credit Y. Zhu).

Figure 22 shows an aggregate of residential buildings visited in Amizmiz. From the exterior they seemed to suffer little damage, but upon visiting the two hybrid RC-masonry buildings on the left we could identify significant damage affecting the internal masonry walls and brittle failure of the reinforced concrete pillars due to interaction with the masonry. The stone masonry building located on the far right has fewer openings and the damage to the stone walls was less significant. We have highlighted the location of parapets in the building that experienced an out-of-plane failure. Lastly, it should be noted that during the mission we deliberately focused on non-collapsed buildings to try to determine failure modes. Close to the epicenter, such as in Talat N’Yaaqoub, approximately 11 km from the epicenter, there were several collapsed masonry and reinforced concrete buildings (Fig. 23), but the extensive damages prevent any analysis of the failure mechanisms, and, therefore, such buildings are not the subject of this report.

## Stone masonry buildings

### Structural characteristics

**Fig. 24:**
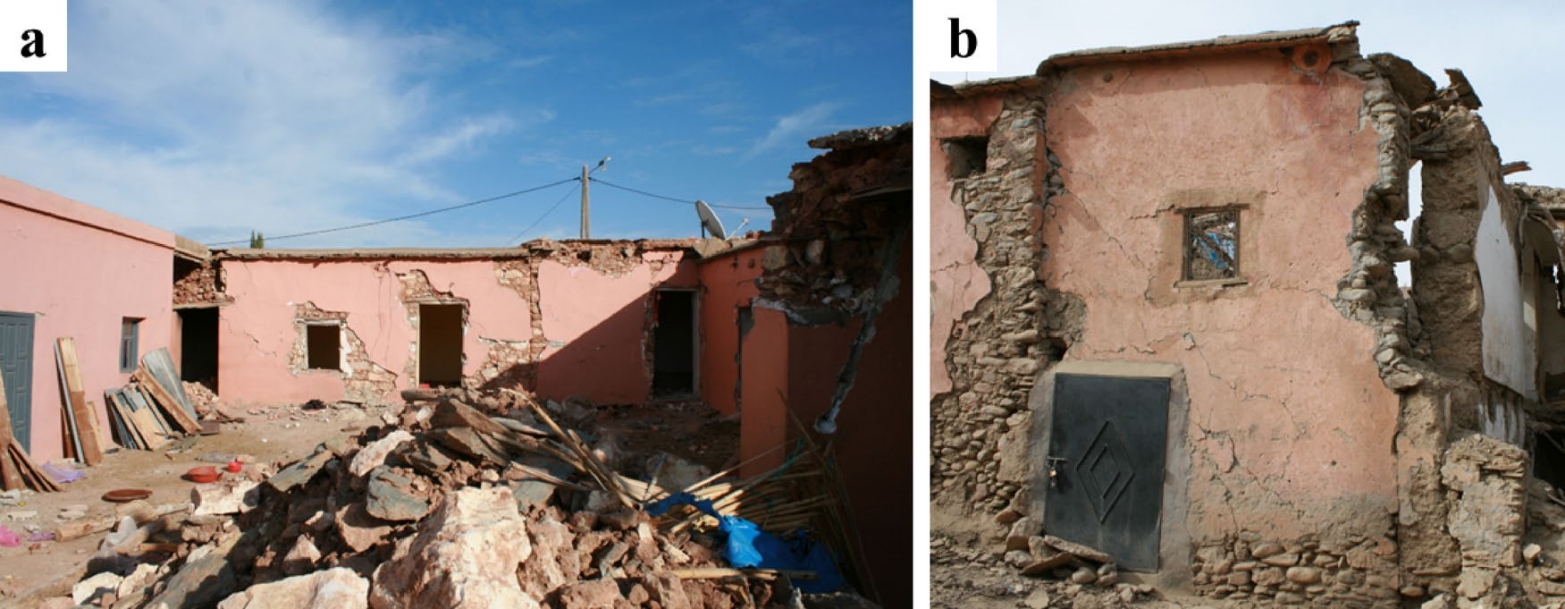
Typical vernacular stone masonry buildings: (**a**) in Tafeghaghte, and (**b**) in Amizmiz (credit S. Saloustros).

**Fig. 25:**
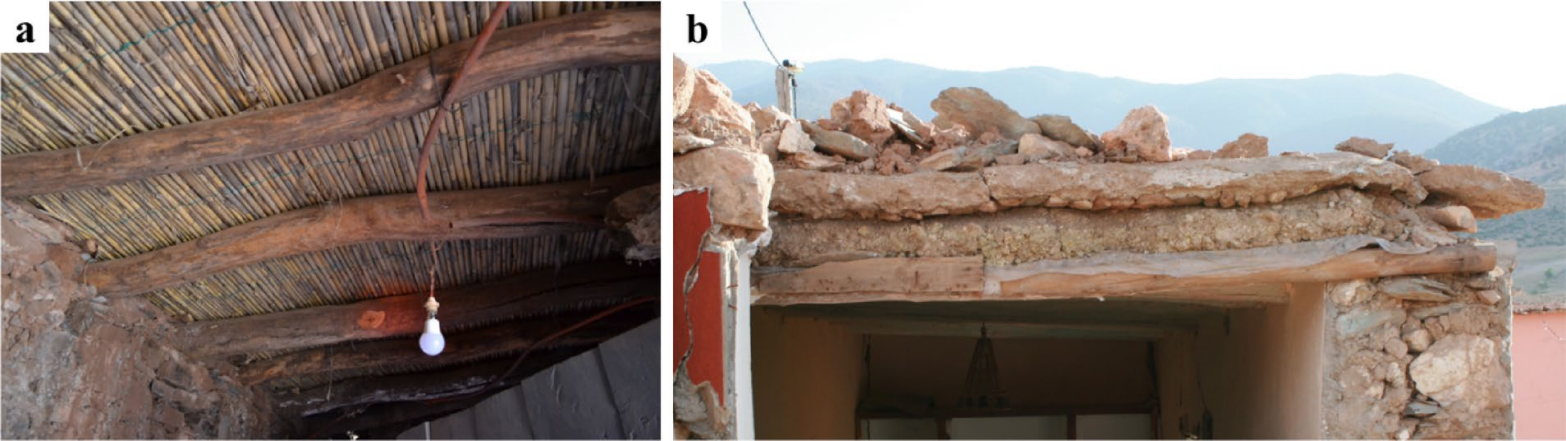
(**a**) Bottom view of the traditional floor from the interior of a building in Toufssirine (credit A. Imtiaz) and (**b**) a section of the floor in a collapsed building in Tafeghaghte (credit S. Saloustros).

Stone masonry residential buildings were common in visited areas close to the epicenter. They typically had one to two stories and were characterized by small spaces and openings with timber used for window and door lintels (see Fig. 24). Stone masonry walls are constructed using multiple leaves with irregularly shaped stones of various sizes, with joints filled using earthen mortar. Inspection of collapsed stone masonry walls indicates a limited number or the complete absence of through-stones connecting the external walls leaves, and the infill was typically composed of small pebbles and earthen mortar (see Fig. 25b).

The construction of stone masonry and earthen structures in Morocco is guided by the Seismic Regulation for Construction in Earth (RPCTerre^28^, decreed by the Ministry of Housing in 2013. This standard indicates methods for the seismic analysis and design and construction details for improving their seismic response. In the surveyed areas, it is estimated that the existing building stock predates the publication of this standard and most of the vernacular buildings lack engineering design. This, coupled with the infrequency of earthquake events in the zone, has resulted in vernacular buildings without structural characteristics that could improve their seismic response, such as good wall-to-wall and floor-to-wall connections and presence of rigid floors.

Figure 25 presents the traditional floor system, which is common in both stone and earthen vernacular buildings. It is comprised of layers of timber and soil materials organized from bottom to top as follows:

- Rounded timber joists spanning in a single direction. The connection with the walls relies solely on friction, and in many cases, the joints extend through the entire wall thickness.
- Secondary timber joists positioned perpendicular to the main joists and placed atop them. These are smaller in diameter, and in several cases, completely absent.
- Wooden mesh positioned atop the timber joists to enclose the open space and provide the support for overlay materials.
- Layer of soil material with a thickness between 0.15 and 0.30 m according to RPCTerre.
- Top finishing using diverse materials, such as stone tiles, earth plaster, or cement mortar.

In many cases, stone buildings exhibited one or more of the following structural alterations: substitution of the traditional floor with a RC or beam-block floor, and incorporation of horizontal (tie-beams, ring beams) and vertical elements (tie-columns) from reinforced concrete. Additionally, vertical extensions with the addition of more floors made from modern materials were also common in the areas we visited.

### Damage in stone masonry buildings

Vernacular stone masonry structures exhibited characteristic damage patterns and failure typologies attributable to their inherent structural vulnerabilities. These included cracking between orthogonal walls resulting from inadequate wall-to-wall connections, wall leaf separation, and out-of-plane collapse due to low quality masonry and the lack of through stones connecting the wall leaves. The heavy and flexible one-way diaphragms, coupled with their deficient wall connections, led to a lack of box-behavior contributing to the local collapse of walls.

**Fig. 26:**
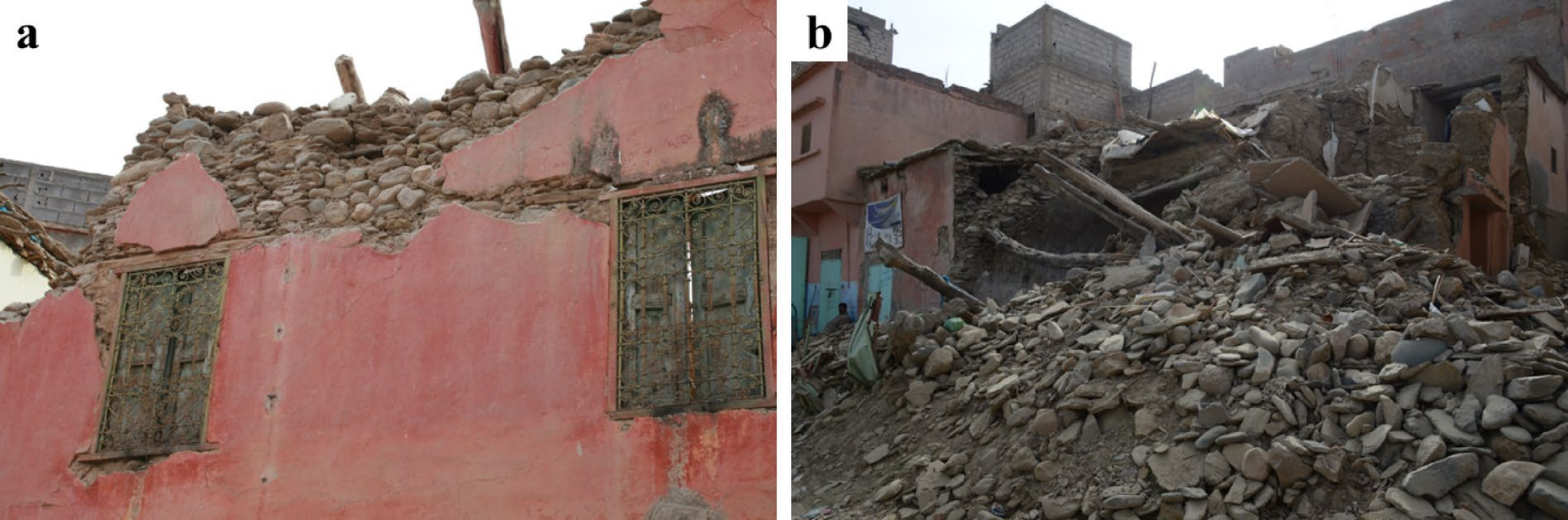
Stone masonry buildings with smooth and rounded stone units in Amizmiz: (**a**) local collapse at the top floor (credit S. Saloustros) and (**b**) global collapse in a corner building (credit Y. Zhu).

The use of stone units sourced from riverbeds was common in the surveyed areas. The rounded shape of these units hampers effective interlocking, while their smooth surface reduces frictional strength^29^. These characteristics, coupled with the low cohesion of earthen mortar in joints, diminish the shear capacity of the walls. Consequently, this resulted in many cases in wall disintegration under the seismic loading, leading to global or partial collapse, as illustrated in Fig. 26.

**Fig. 27:**
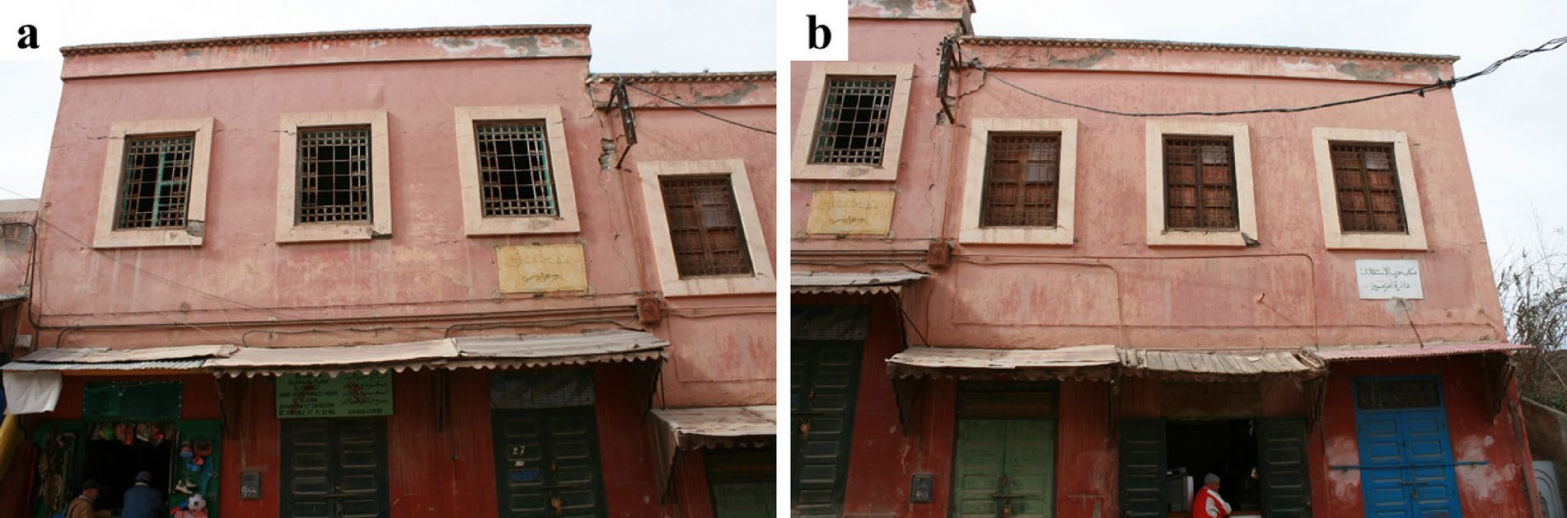
Flexural cracking at the top and bottom of piers at the top floor in two adjacent stone masonry structures in Amizmiz (credit S. Saloustros).

**Fig. 28:**
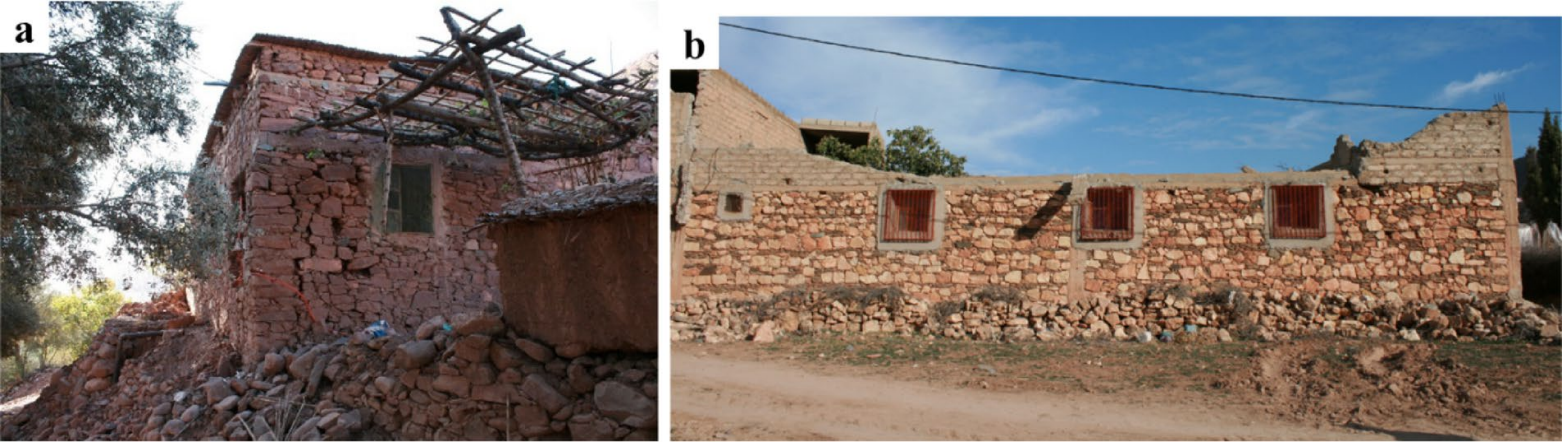
Examples of buildings with structural and construction characteristics that prevented collapse in: (**a**) Tikioute and (**b**) Tafeghaghte (credit S. Saloustros).

On the other hand, structures with geometrical regularity and good masonry quality experienced less damage and collapses. Figure 27 presents two neighbouring stone masonry buildings with regular distribution of openings at Amizmiz, and masonry made from irregular stones with rough surfaces. The only damage visible from the exterior in both buildings concerns thin horizontal flexural cracks at the top and bottom of the piers of the top floor. A similar example showing how good construction practices can improve the seismic response of vernacular stone masonry buildings are shown in Fig. 28. The building in Fig. 28 (a), while presenting important damage, is one of the few non-collapsed stone masonry buildings in Tikioute. The structure exhibits structural and construction characteristics absent in the neighbouring buildings: (i) the masonry units display greater regularity with rough surfaces and rectangular shapes, (ii) the earthen mortar joints are thin, and (iii) a concrete tie-beam encircles the building at the level of the window openings. The building of Fig. 28 (b) is one of the few buildings of Tafeghaghte that remained visibly intact, exhibiting minimal visible damage of the stone masonry walls from the exterior. For this case as well, stone masonry consists of approximately rectangular units with thin mortar joints presenting a good horizontal alignment. These construction characteristics are indicative of a better stone masonry quality^30^.

**Fig. 29:**
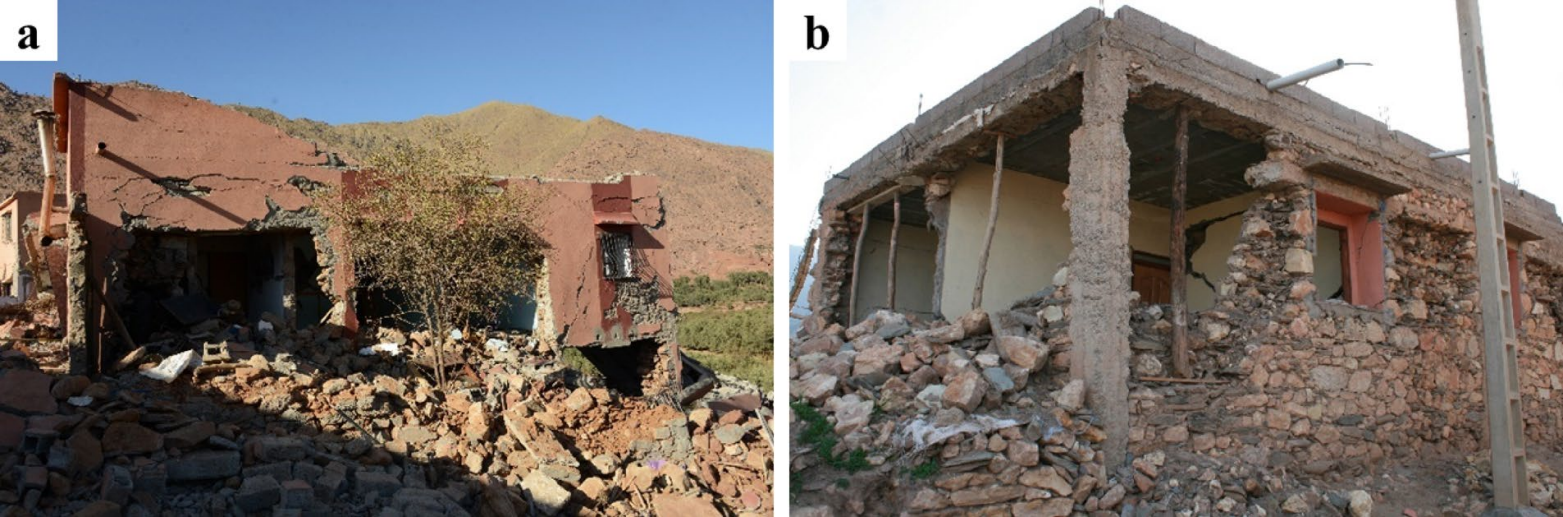
Local collapse of stone masonry walls in buildings with reinforced concrete piers, beams, and floors in: (**a**) Talat N’Yaaqoub (credit Y. Zhu) and (**b**) Tafeghaghte (credit S. Saloustros).

In the villages of Tafeghaghte and Talat N’Yaaqoub, numerous buildings displayed a mixed structural system comprising stone masonry walls along with reinforced concrete piers, ring beams, and a modern floor system. As illustrated in Fig. 29, these structures exhibited significant damage, characterized by the partial or complete out-of-plane collapse of the stone masonry walls situated between the reinforced concrete elements. This suggests an independent structural response of RC and stone masonry elements contrary to what is expected in confined masonry structures. The collapse of the walls in these two cases indicates that the floors are supported primarily by the reinforced concrete piers, resulting in a lack of stabilizing vertical force exerted on the walls. The out-of-plane partial or global collapse of the walls (Fig. 29b) was facilitated by the poor quality of stone masonry, which lacked through stones connecting the wall leaves.

**Fig. 30:**
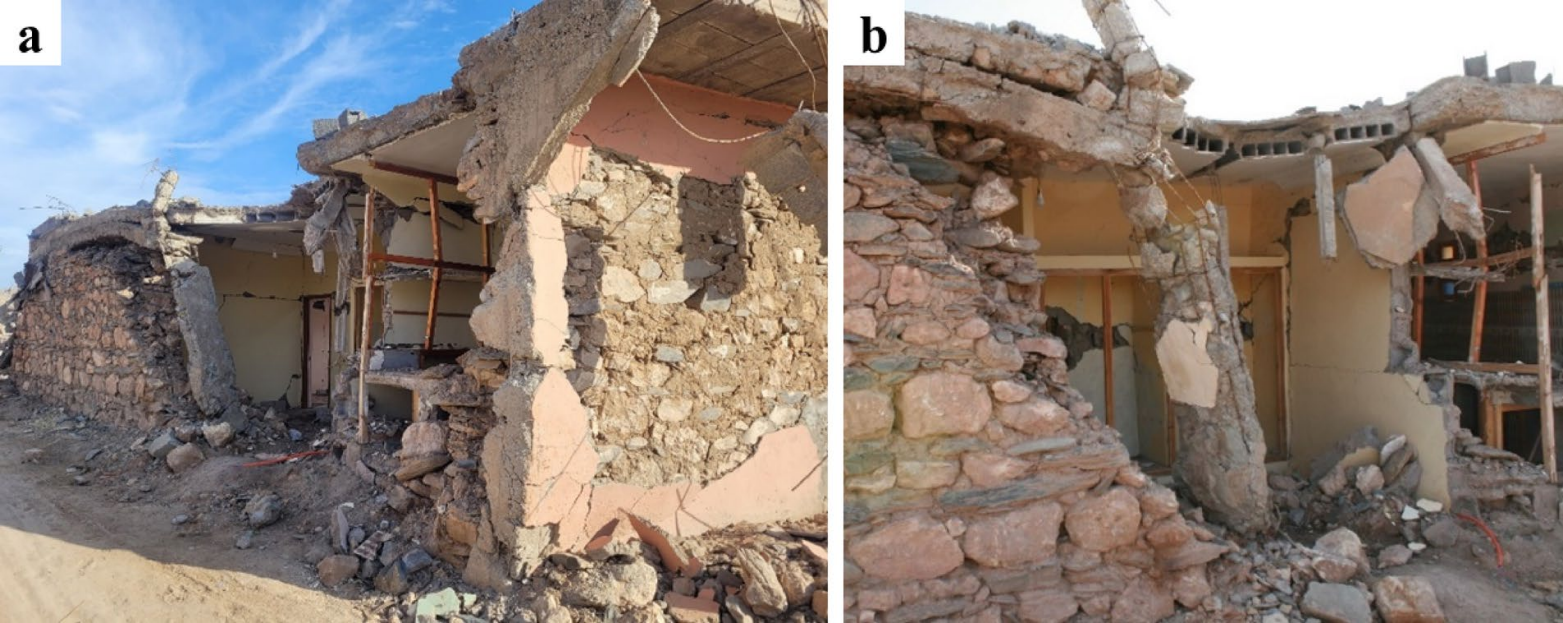
Partial collapse of a stone masonry building with a new beam-block floor in Tafeghaghte. (**a**) The ring beam at the top was completely disconnected from the supporting walls along its whole length, failing to provide the desired box-behavior (credit E. Lattion). (**b**) Closer view of the ring-beam with evidence the presence of large stone units within the reinforcement (credit S. Saloustros).

One of the most common deviations from the traditional structural system of masonry buildings was the substitution of the traditional floor system with a beam-block arrangement. The presence of a modern floor system did not prevent the collapse of structures with poor-quality masonry. Figure 30 shows the partial collapse of the façade walls in a stone masonry building with a new beam-block floor in Tafeghaghte. The top part of the wall in contact with the ring beam was completely disintegrated, indicating that poor-quality masonry cannot ensure sufficient connection with the ring beam. The ring beam itself was of poor quality, as evidenced by the presence of several large stones between the reinforcement. The detachment of the ring beam from the floor system suggests that the two elements were cast separately, failing to establish a connection of the diaphragm with the walls.

**Fig. 31:**
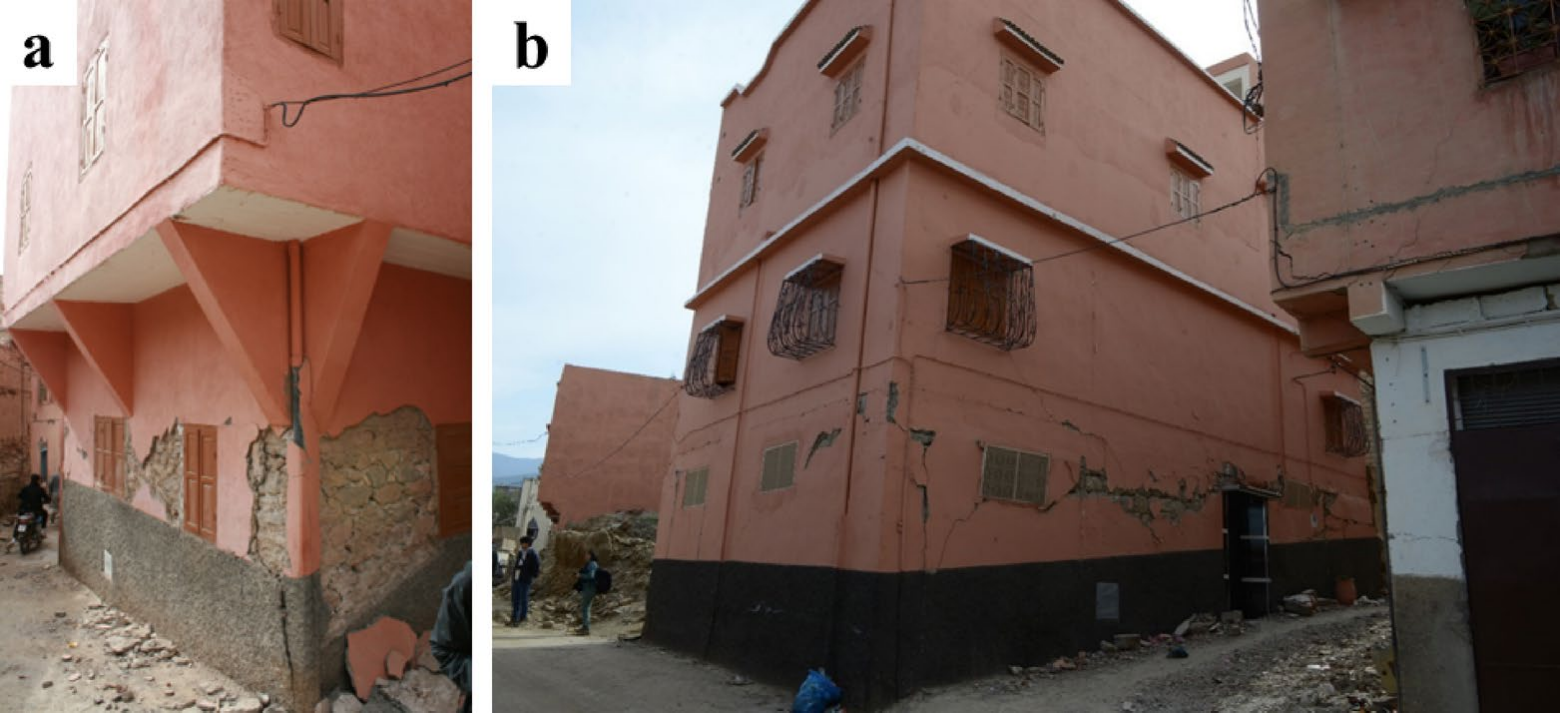
Buildings with stone masonry at the (**a**) ground floor (credit S. Saloustros) and (**b**) vertical floor extensions (credit Y. Zhu) with modern block masonry.

In Amizmiz, several of the stone masonry structures have undergone vertical extensions by adding one or more stories. In most cases, the walls of the new stories are constructed using modern construction typologies, such as confined block masonry. Figure 31 illustrates two examples of such structures. In these cases, the increased vertical load due to the floor addition led to diagonal shear cracking at the ground floor walls, although the structures did not collapse.

**Fig. 32:**
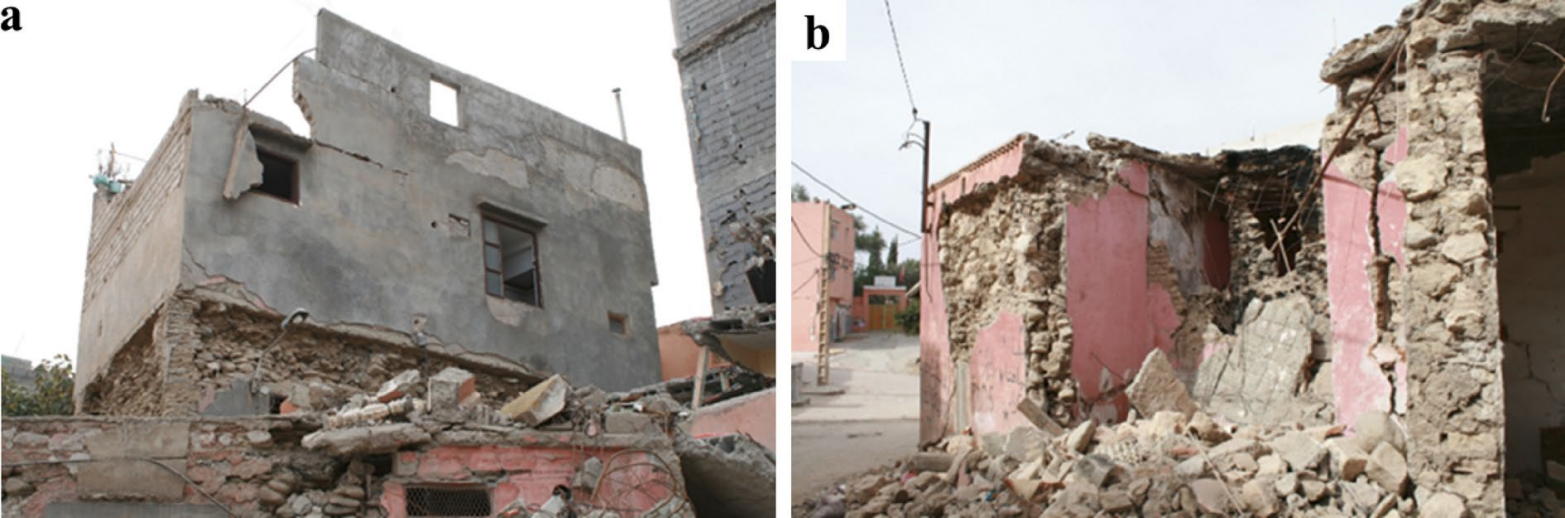
Failure of external wall leaves in (**a**) a building with vertical extensions and (**b**) a single-story stone masonry building in Amizmiz (credit S. Saloustros).

Figure 32 (a) shows a stone masonry building with a vertical extension with more severe damage. The misalignment between the top floor’s corners and those of the ground floor suggests a weak connection between the existing structure and the new addition, leading to the upper level sliding atop the stone masonry. The stone masonry exhibits an out-of-plane failure of the external wall leaf, likely due to a combination of poor connection between the wall leaves and an eccentric application of vertical loads, leaving the external leaf unsupported. This type of local wall failure was commonly observed in most stone masonry structures (see Fig. 32b).

## Earthen structures

### Structural characteristics

Rammed earth and adobe masonry are two construction techniques used for building load-bearing walls made from soil in the surveyed areas. Adobe structures were mainly observed in Toufssirine and Marrakech, i.e. in a distance above 40 km from the epicenter. Rammed earth structures were more prevalent in the rest of the surveyed areas closest to the epicenter.

**Fig. 33:**
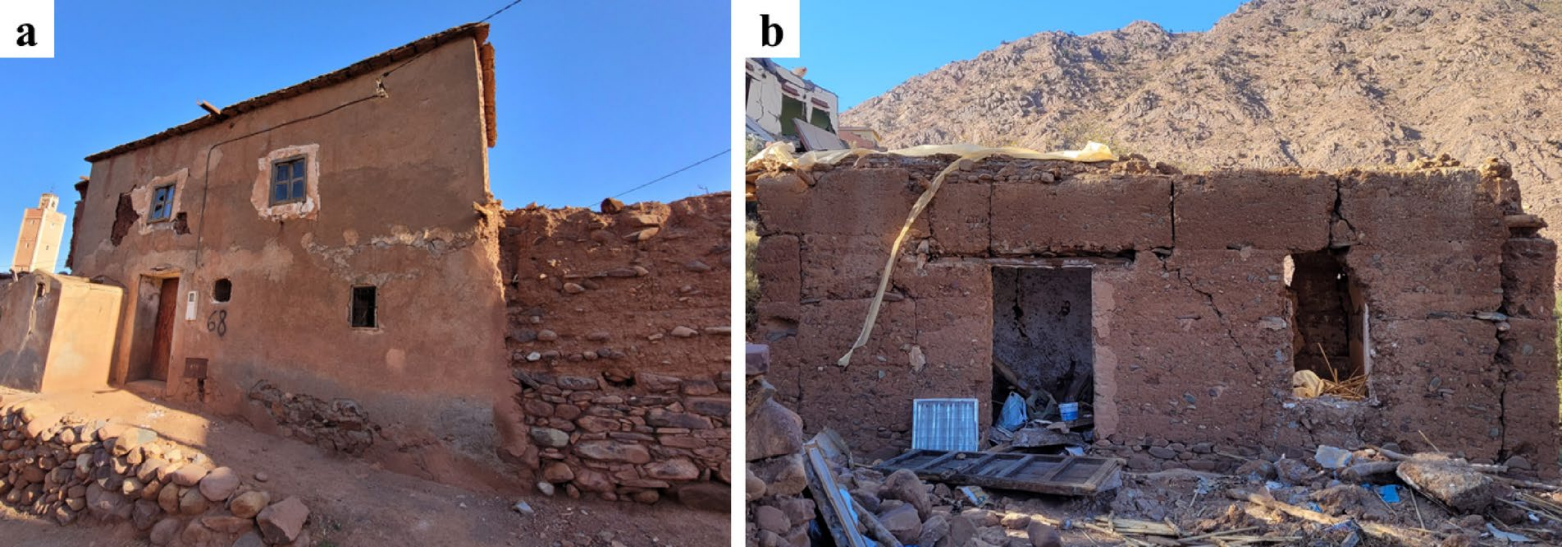
Examples of buildings made from earthen materials: (**a**) two-story earthen building in Toufssirine and (**b**) single-story rammed earthen building in Talat N’Yaaqoub (credit E. Lattion).

**Fig. 34:**
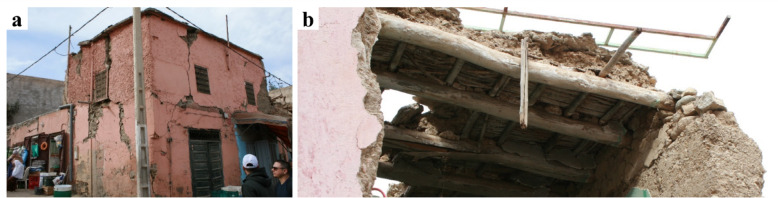
(**a**) Typical two-story building with a stone masonry at the ground floor and rammed earth walls at the first floor in Amizmiz. Cracking at the top floor goes through the interface between neighboring rammed earth blocks (credit S. Saloustros). (**b**) Close-up in a traditional floor system in a partially collapsed building in Amizmiz connected to the rammed earth walls through a stone masonry layer (credit S. Saloustros).

Typical vernacular earthen buildings consist of one to two stories and are characterized by small spaces and openings (Fig. 33). The lower part of the ground floor is typically made of stone masonry to protect the earthen materials from rising damp (Fig. 33a). In Amizmiz, several buildings were constructed with stone masonry on the ground floor and rammed earth walls on the upper floor (Fig. 34a). Earthen buildings were characterized by large material heterogeneity, with a combination of construction materials and techniques, such as rammed earth with adobe, or rammed earth with stone masonry. Window and door lintels were typically made from timber.

Floors in earthen buildings are made using the traditional floor system consisting of timber and soil, as already detailed in the section of stone masonry buildings. In several cases, a layer of stone masonry was observed at the level of the floor (Fig. 34b). Unlike stone masonry structures, vernacular earthen structures in the surveyed areas did not present significant structural alterations with the introduction of modern construction materials. RPCTerre (2011) provides design procedures and construction practices for earthen structures under seismic actions. Most earthen buildings in the visited areas, like stone masonry ones, date before the implementation of RPCTerre (2011) and they lack the construction and structural characteristics that could improve their seismic performance by improving the box behavior.

### Damage in earthen buildings

**Fig. 35:**
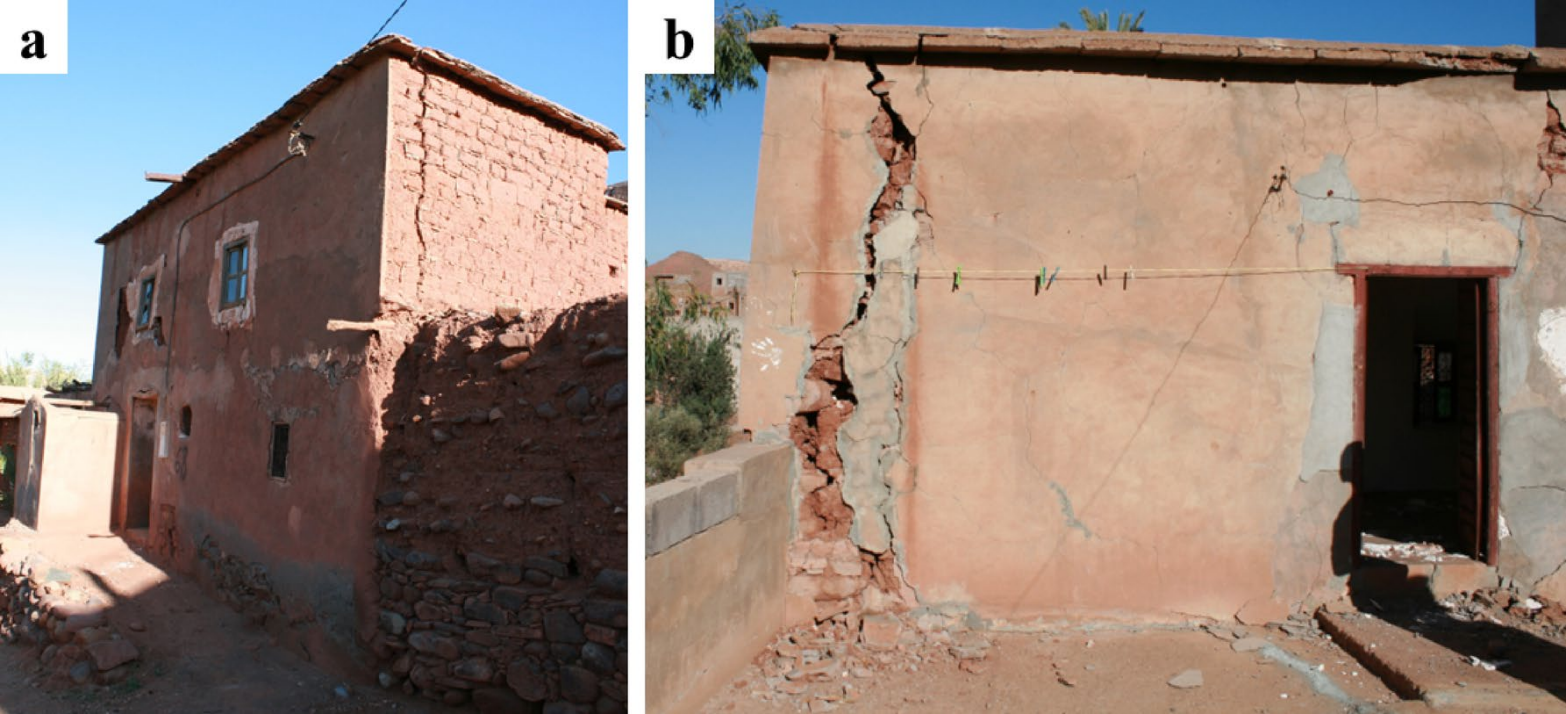
Vertical cracking between orthogonal walls in: (**a**) adobe, and (**b**) a rammed earth building in Toufsirrine (credit S. Saloustros).

The separation of orthogonal walls through vertical cracking was one of the most observed damage patterns in the surveyed earthen buildings. Figure 35 illustrates two cases of vertical cracking between orthogonal walls in adobe and rammed earth buildings. This damage typology indicates that the tensile strength of the earthen units was insufficient to transfer the horizontal load from the wall loaded out of its plane to its orthogonal one. In the visited areas, we did not identify a systematic use of timber or other materials to improve wall-to-wall or wall-to-floor connections and allow therefore a force redistribution between orthogonal walls during the seismic loading.

Construction joints between different materials or between rammed earth blocks were observed to be a weak point in most of the surveyed vernacular earthen buildings. When interlocking at corners and the tensile and shear strength of rammed earth, and adobe units and earthen mortar joints were enough to avoid vertical cracking at the intersection of orthogonal walls, cracking localized at construction joints further away from the corner (see Fig. 34a).

**Fig. 36:**
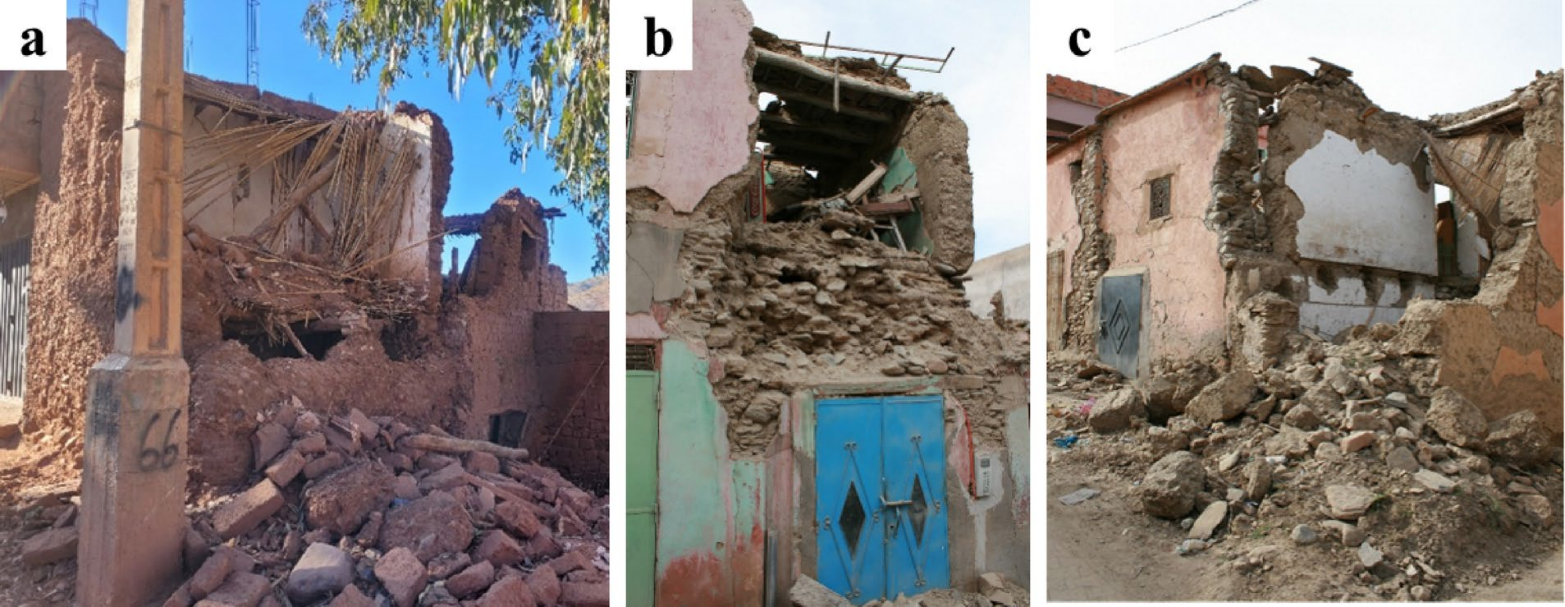
Out-of-plane collapses of walls in earthen buildings: (**a**) rammed earth and adobe walls and floor collapse in neighboring buildings in Toufssirine (credit E. Lattion), (**b**) rammed earth façade in Amizmiz. The stone masonry ground floor exhibits an out-of-plane collapse of the external wall leaf (credit S. Saloustros), and (**c**) collapse of a rammed earth building in Amizmiz. Poor interlocking with neighboring building and with orthogonal walls (credit S. Saloustros).

**Fig. 37:**
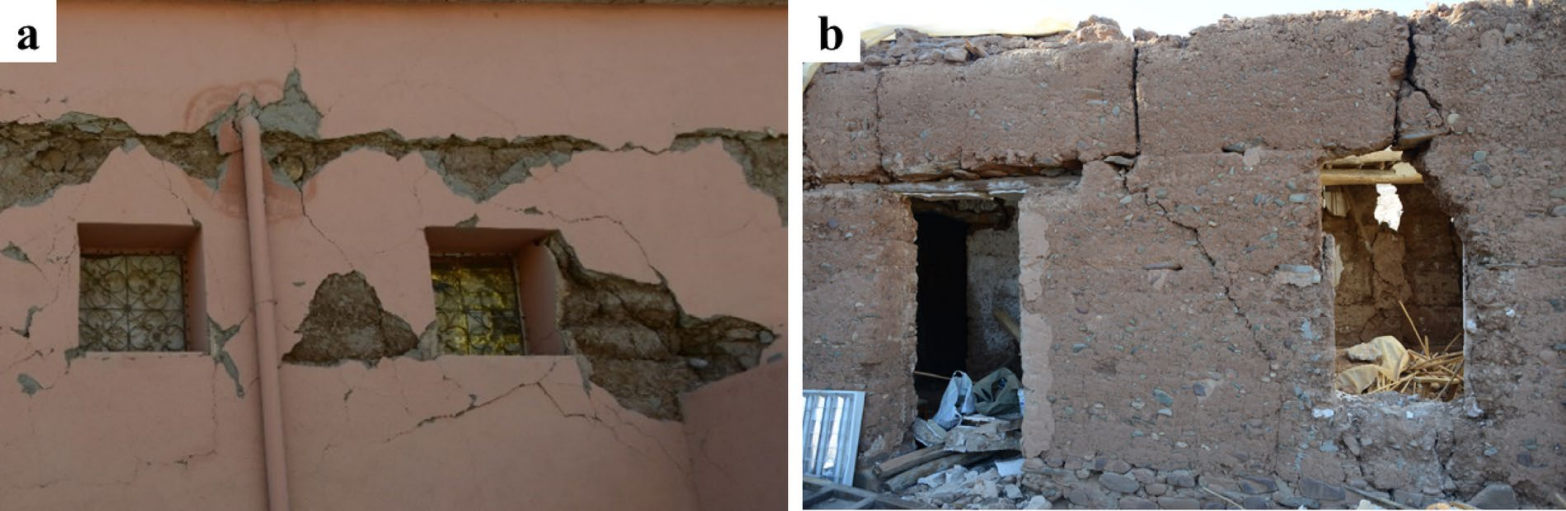
In-plane shear cracking: (**a**) in short walls between opening in a rammed earth building in Imgdal, and (**b**) in wide pier of a rammed earth building in Talat N’Yaaqoub (same building as in Fig. 30b) (credit Y. Zhu).

Wall overturning was a common failure pattern observed in earthen structures in the visited areas, as shown in Fig. 36. This pattern frequently occurred in façade walls parallel to the direction of the floor joists, suggesting that the absence of vertical stabilizing force from the vertical floor loads, coupled with the lack of interlocking between orthogonal walls, heightened their vulnerability to out-of-plane movement. On the other hand, only a few buildings presented in-plane diagonal cracking. Figure 37 (a) illustrates diagonal shear cracking in short columns between window openings in a building in Imgdal. Figure 37 (b) (see also Fig. 33) shows a single-floor rammed earth building in Talat N’Yaaqoub, which exhibits diagonal cracking in a wide pier.

## Observed damage in a typical building: a case study

**Fig. 38:**
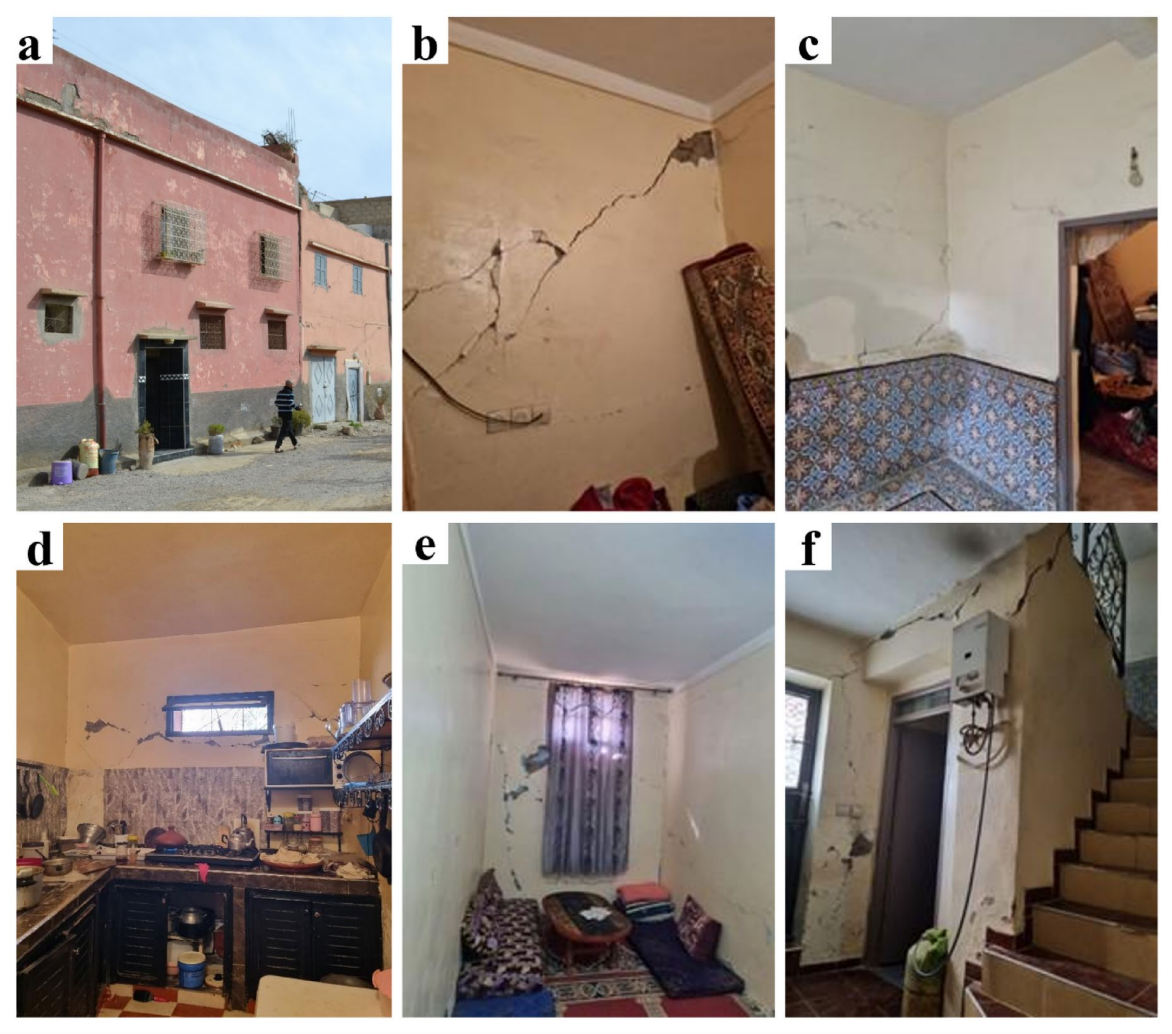
(**a**) The evaluated building and (**b**–**f**) observed damage in different parts of the ground floor (credit G. Cortés).

One of the mission’s objectives was to test the Swiss post-seismic building evaluation form (OFPP 2023) for a sample of buildings. The form’s goal is to assign a damage grade and determine the building’s habitability. The assessment includes sections for building identification (location, age, occupancy), typology classification, seismic vulnerability assessment, identification of the level of damage undergone by the structural and non-structural elements and collapse risk assessment. An example of a surveyed building in Amizmiz is shown hereafter (Fig. 38). According to the owner, initial construction of this building began in 1975 and extensions were added later. Positioned on a corner plot, the building rises over two floors with an additional room and terrace on the third level, and no basement, the surface area and height measuring approximately 12 m × 12 m and 9 m, respectively. This house was inhabited by a family of 6 people.

The construction of the upper floors consisted of concrete frames with masonry infills. Since the walls were plastered, it was difficult to determine whether this was a confined masonry or a frame structure with masonry infill. The type of masonry used varied, from stone at ground level to brick at upper levels. The concrete floor slabs provided relatively rigid diaphragms. No horizontal irregularity was observed, but vertical irregularities were caused by the offset of a façade wall above the ground floor and the presence of non-continuous vertical elements such as walls and reinforced concrete columns was present. The materials used were likely of poor quality, especially the stone masonry used in the lower parts of the building. In addition, the connection with the adjacent building seemed of poor quality.

Although exterior walls seemed undamaged or lightly damaged from the outside, more significant damage, in the form of cracks, was observed from the interior of the building. Figure 38 (b–f) shows the level of damage observed on the ground floor. It corresponds to a level of damage of degree 3 according to the European Macroseismic Scale EMS-98^31^. Grade 3 describes buildings that have suffered substantial to significant damage, with large and extensive cracks observed in most walls. Given the construction methods employed, no distinct non-structural element was observed, while all partitions formed part of the structural system. The adjacent building (Fig. 38a) also suffered significant damage, including partial collapse of the upper level, but this did not appear to pose a significant threat to the building examined. The residual capacity of the building has been significantly reduced and therefore, if another significant aftershock occurs, this building would be susceptible to damage of Grade 4 or greater (EMS 98).

## Discussion and conclusion

Observations of the SGEB team from the reconnaissance mission of the 2023 Al Haouz underscore the complex interplay of geological, topographical, and site-specific factors influencing the seismic hazard and potentially amplifying ground motions. The resulting damage inflicted on communities, particularly those situated near mountain slopes and within sedimentary basins, highlights the importance of research and monitoring efforts in site-specific studies, examining subsurface conditions, seismic velocities, and local geological structures through geophysical and seismological data. Such studies can guide the design and construction of buildings and infrastructure, playing a crucial role in bolstering communities’ resilience against earthquakes.

Our damage observations in the affected areas deliberately focused on non-collapsed buildings to determine the failure modes. In Marrakech, contemporary buildings, including modern hotels and new residential developments, are generally reinforced concrete structures built using modern construction techniques. Hence, they were largely unaffected while the ancient Medina of Marrakech suffered partial damage. The implementation of emergency shoring around mosque structures and other buildings in Medina suggests that out-of-plane tilting mechanisms were induced by the earthquake ground motion.

In rural areas of High Atlas Mountain, hybrid concrete-masonry buildings incorporate multiple lateral load-resisting systems, often due to modifications and expansions. These structures have complex responses to seismic shaking due to significant strength and stiffness discontinuities in their load transfer mechanisms, especially at material junctions. This increases their seismic vulnerability. Additionally, the low quality of local materials (concrete and bricks) exacerbated damage.

Stone masonry buildings in surveyed rural areas were among the most severely impacted by the earthquake. The main causes of damage and collapse were the lack of box-like behavior and the use of poor-quality masonry, characterized by irregular, smooth-surfaced stones, thick earthen-mortar joints, and weak wall-to-wall and wall-to-floor connections. These factors compromised wall integrity, leading to partial or total collapses. More resilient structures used regular stone units, thin mortar joints, and sometimes incorporated tying elements. However, structural modifications, such as adding reinforced concrete elements and modern floor systems, often failed to prevent collapses in buildings made from poor-quality stone masonry.

Earthen structures also exhibited significant damage, often resulting in partial wall and floor collapses. The main cause was the lack of box-like behavior due to inadequate wall connections and flexible diaphragms. The common failure mechanism was out-of-plane wall overturning with separation of orthogonal walls.

To improve building practices and community preparedness in Moroccan seismic regions, especially urban centers like Marrakech, a multifaceted approach is essential. First, strengthening the enforcement of the seismic code for existing and new structures could further enhance the safety and resilience of buildings. The Al Haouz earthquake of 2023 exposed systemic deficiencies, such as, structural collapse linked to poor material quality and non-compliance with code-specified seismic engineering design. Retrofitting programs have the potential to significantly enhance safety of highly vulnerable structures, such as schools, hospitals, and older residential blocks. These efforts can be even more effective when paired with financial incentives that support homeowners in making improvements. Strengthening material quality control on construction sites can also contribute to improving seismic safety. Providing training in seismic-resistant detailing offers a valuable opportunity to build local expertise, especially among informal or semi-skilled workers. At the community level, public education campaigns and local preparedness plans, including evacuation drills and damage response training, can play an important role in building seismic resilience. Collaborative efforts between universities, municipal authorities, and engineering bodies can further support ongoing research, risk assessment, and resilience building efforts.

From our observations, we learned that clear risk communication can keep communities informed and builds trust. Community healing activities aid trauma coping, and inclusive recovery strengthens bonds. Fast solutions for temporary housing and resilient rebuilding are essential. Enforcing earthquake-resistant design in rural areas is challenging due to cost barriers, but training local builders in these practices ensures minimum safety standards. Combining modern earthquake-proofing with traditional techniques fosters cultural continuity and resilience. Involving local communities in decision-making ensures recovery efforts meet their needs and leverages local knowledge, enhancing rebuilding effectiveness and providing a holistic recovery approach.

## Data Availability

The seismic data used in this study was downloaded by querying through the web services of the International Federation of Digital Seismograph Networks (FDSN) at https://www.fdsn.org/webservices/ (last accessed February, 2024). The data centers that hosted the seismic data are cited in Table 1 of the article and listed in the references. All photos used in this work have been taken by the authors of this article during the post-seismic mission in Morocco and the repository is available at https://doi.org/10.5281/zenodo.11066140 (last accessed May, 2024).
